# Evidence for Host-Bacterial Co-evolution via Genome Sequence Analysis of 480 Thai *Mycobacterium tuberculosis* Lineage 1 Isolates

**DOI:** 10.1038/s41598-018-29986-3

**Published:** 2018-08-02

**Authors:** Prasit Palittapongarnpim, Pravech Ajawatanawong, Wasna Viratyosin, Nat Smittipat, Areeya Disratthakit, Surakameth Mahasirimongkol, Hideki Yanai, Norio Yamada, Supalert Nedsuwan, Worarat Imasanguan, Pacharee Kantipong, Boonchai Chaiyasirinroje, Jiraporn Wongyai, Licht Toyo-oka, Jody Phelan, Julian Parkhill, Taane G. Clark, Martin L. Hibberd, Wuthiwat Ruengchai, Panawun Palittapongarnpim, Tada Juthayothin, Sissades Tongsima, Katsushi Tokunaga

**Affiliations:** 1 Department of Microbiology, Faculty of Science, Mahidol University, Rama 6 Road, Bangkok, Thailand; 2 National Centre for Genetic Engineering and Biotechnology, National Science and Technology Development Agency, Phahonyothin Road, Pathumthani, Thailand; 3 Department of Medical Sciences, Ministry of Public Health, Tiwanon Road, Nonthaburi, Thailand; 4 TB-HIV Research Foundation, Chiangrai, Thailand; 5 Fukujuji Hospital, Japan Anti-tuberculosis Association (JATA), Kiyose, Japan; 6 Research Institute of Tuberculosis, JATA, Kiyose, Japan; 7 Chiangrai Prachanukroh Hospital, Ministry of Public Health, Chiangrai, Thailand; 8 Department of Human Genetics, Graduate School of Medicine, the University of Tokyo, Tokyo, Japan; 9 London School of Hygiene and Tropical Medicine, London, UK; 10 Welcome Trust Sanger Institute, Hinxton, Cambridge, UK

## Abstract

Tuberculosis presents a global health challenge. *Mycobacterium tuberculosis* is divided into several lineages, each with a different geographical distribution. *M. tuberculosis* lineage 1 (L1) is common in the high-burden areas in East Africa and Southeast Asia. Although the founder effect contributes significantly to the phylogeographic profile, co-evolution between the host and *M. tuberculosis* may also play a role. Here, we reported the genomic analysis of 480 L1 isolates from patients in northern Thailand. The studied bacterial population was genetically diverse, allowing the identification of a total of 18 sublineages distributed into three major clades. The majority of isolates belonged to L1.1 followed by L1.2.1 and L1.2.2. Comparison of the single nucleotide variant (SNV) phylogenetic tree and the clades defined by spoligotyping revealed some monophyletic clades representing EAI2_MNL, EAI2_NTM and EAI6_BGD1 spoligotypes. Our work demonstrates that ambiguity in spoligotype assignment could be partially resolved if the entire DR region is investigated. Using the information to map L1 diversity across Southeast Asia highlighted differences in the dominant strain-types in each individual country, despite extensive interactions between populations over time. This finding supported the hypothesis that there is co-evolution between the bacteria and the host, and have implications for tuberculosis disease control.

## Introduction

The burden of tuberculosis is high in developing countries in Asia, with 56% of all global cases found in five countries: India, Indonesia, the Philippines, Pakistan and China^1^. With tremendous efforts, some new drugs have recently been developed, but there has still been limited success in developing new effective tuberculosis vaccines. Thus a better understanding of the interactions between *M. tuberculosis* and its host is urgently needed.

*M. tuberculosis* lineages strongly associate with the geographic location, ethnicity and ages of the hosts^2^, which could be due to the founder effect or co-adaptation between the bacterial and host population, favouring their stable coexistence^3,4^. The latter hypothesis was supported by the sympatric association between bacterial lineages and patients’ origins^5^ which was weakened in HIV-infected patients^6^. The global population structure of *M. tuberculosis* lineage 4 further supported the co-evolution hypothesis^7^. Moreover, some candidate gene studies revealed an association with diseases caused by only some lineages^8^. Recently, genome-wide association studies (GWAS) successfully identified the HLA-DRB1*09:01 and CD53 genes associated with tuberculosis when the bacterial genotypes were considered^9,10^.

Other aspects of genotype-phenotype associations have also been studied, particularly for lineages 2 and 4. The association varied between sublineages. The sublineages of the Beijing strains were associated with differential survival rates, virulence and responses to vaccine^2,11–13^.

Unfortunately, there have been few studies on the phenotypes of lineage 1 (L1). L1, as defined by SNP typing, is congruent with the Indo-oceanic strains, defined by LSP (large sequence polymorphisms)^14^ and almost completely congruent with the EAI (East African Indian) strains, defined by spoligotyping. The EAI strains were commonly reported in countries around the Indian Ocean^15,16^ and associated with higher ages of patients^17^. Pulmonary tuberculosis caused by L1 strains was more likely to be sputum negative and had a higher sputum conversion rate at two months after treatment^18^. L1 was less associated with drug resistance than L2^19,20^. They were occasionally reported to associate with extrapulmonary tuberculosis^21^. In general, L1 isolates induced stronger cytokine responses and grew slower in macrophage culture^22,23^.

Studies of L1 are essential for the End TB strategy^24^. It is endemic in 11 of 30 high- burden countries. Some bacterial isolates, such as the “Asian human” type, from India and East Africa had distinct properties^25^ and were hypothesized to provide an explanation for the lack of efficacy of the BCG vaccine in the monumental clinical trial in Tamil Nadu, Southern India^26^. The described phenotypes might associate with L1 strains.

Recently, Coll *et al*. proposed classification based on a whole-genome single nucleotide variant (SNV) phylogenetic tree and provided a corresponding barcoding scheme^27^. While there have been many WGS studies of lineage 2 strains, studies of L1 strains are rather limited.

Comparing epidemiological findings between countries requires a reliable and high-resolution classification based on the bacterial genetic relationship. EAI isolates are classified by spoligotyping into “clades”, such as EAI1-EAI8. However, it is not clear whether the “clades” are monophyletic. There are “orphan” spoligotypes that are not yet assigned to any clades while assignment of some spoligotypes may be problematic. Correlation of the SNV phylogenetic tree to spoligotypes would provide better insights into the phylogeography of L1.

In this study, the genomes of 480 isolates of *M. tuberculosis* L1 in Chiangrai, the northernmost province of Thailand, were sequenced and revealed significant diversity. Analysis of WGS was done to refine the SNV phylogenetics of L1, verify spoligotype clades and provide mapping between spoligotypes and the SNV phylogenetic tree. This finding provided a basis to map the distribution of predominant sublineages of L1 in various countries in Southeast Asia. Our results suggested an association between predominant sublineages and geography and ethnicity, despite a history of extensive interactions between various ethnic groups, supporting the hypothesis that there is some level of co-adaptation between *M. tuberculosis* L1 and human hosts.

## Results

### Classification of *M. tuberculosis* L1

Among 1174 successfully sequenced *M. tuberculosis* isolates, 480 were initially identified by LSP as belonging to the Indo-Oceanic family and had the 330 SNPs, previously reported to be specific to L1^27^ (Supplementary Table S1). The following results describe phylogenetic analysis of WGS information from the 480 isolates. The resulting sublineages were then compared to the sequences of the Direct Repeat (DR) region obtained from WGS and examined by spoligotyping. The DR region contains a maximum of 69 DR sequences, interposed by 68 Direct Variable Repeats (DVRs). Variations between strains are due to the absence of some DVRs. The information on the absence of each of the 68 DVRs was extracted from WGS. Standard spoligotyping determines the absence of 43 selected DVRs and usually reports results for each isolate as a 15-digit octal code or equivalently designated spoligotype international type (SIT).

A total of 41,157 SNVs were identified, with 22,491 SNVs, each present in only a single isolate. The total identified SNVs were more than in the previous reports (Supplementary Table S2). L1 is the most genetically diverse lineage of *M. tuberculosis*^2,27^. Its mean pairwise SNV distance (MPSD) in this study was 645.6 (range 0–965), while that of the globally reported L1 isolates was 730^2^, indicating high heterogeneity of L1 in Chiangrai. This finding might be related to the knowledge that Chiangrai is an ancient settlement originally with Austroasiatic language-speaking people and subsequently replaced by people who speak the Tai-Kadai language family^28,29^. It was historically controlled by several tribal kingdoms that resided now in Thailand, Myanmar and Lao. Chiangrai is home to several hill tribes and recent Chinese immigrants. Its modern roles as a transport hub and a major tourist attraction site lead to rapid urbanization and migration from throughout Thailand and other countries.

The spoligotypes of 410 (85%) isolates were classified as EAI strains, while those of the other 66 isolates were not previously described^30^. However, one and three isolates had spoligotypes of 777777777773771 (SIT100) and 777777777777771 (SIT523) respectively. Neither pattern belongs to the EAI spoligotypes even though all four had the genetic markers of L1 (TbD1+ and RD239−). Isolates with SIT100 belonging to L1 have been previously reported^31^.

Analysis of all 68 DVR segments revealed that all EAI isolates and unclassified isolates lacked DVR39-42, DVR44, known characteristics of EAI, and DVR48, which is not used for standard spoligotyping. This finding indicated that all unclassified isolates belonged to the EAI family. Some isolates had all the other 62 DVRs, indicating that the most recent common ancestor of the EAI group (EAI-MRCA) carried the deletion of only DVR39-42, 44 and 48.

### Whole-genome SNV phylogenetic tree of L1

**Figure 1:**
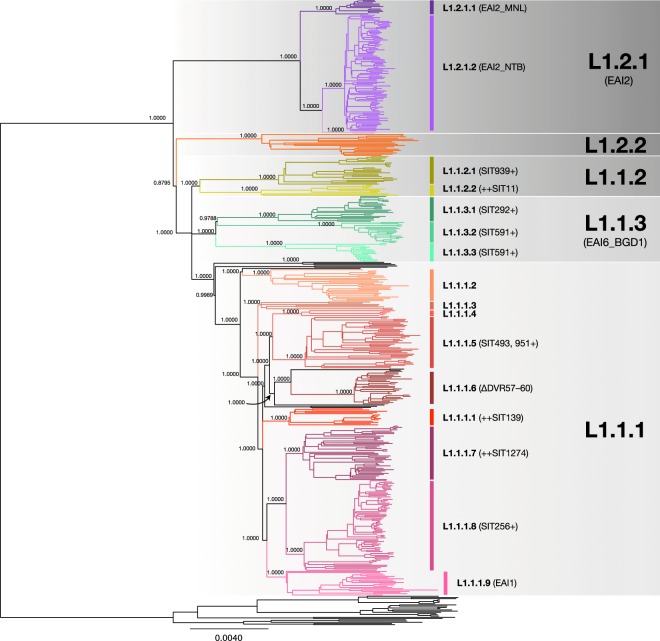
The phylogenetic tree of 480 *M. tuberculosis* L1 isolates from Chiangrai, constructed by the Bayesian inference method. The major subgroups were labelled on the rightmost. L1.1.1.1 were the same group as reported by Coll *et al*. The sublineages L1.1.1.2–L1.1.1.9, labelled on the right, were named sequentially according to their positions in the tree. Some interesting spoligotypes are shown after the sublineage names. The SIT number followed by the + sign indicates that the shown SIT contributed to the majority of the sublineage. The ++ sign before the SIT number indicates that the spoligotype was only a small fraction of the sublineage. The deletion of DVR57-60 was specific to L1.1.1.6 but was not revealed by spoligotyping because the DVRs were not used for standard spoligotyping.

Coll *et al*. studied 1601 globally collected WGS deposited in public databases, classifying 121 L1 isolates into five major groups, 1.1.1–1.1.3 and 1.2.1–1.2.2^27^. The phylogenetic trees of the 480 isolates constructed by Bayesian inference (Fig. 1) and maximum likelihood (Supplementary Fig. S1) methods were very similar to each other and mostly conformed to Coll’s report. They revealed deep branching into three major groups, corresponding to L1.1, L1.2.1 and L1.2.2, as indicated by the similarity of many sublineage-specific SNPs in Coll’s work and in our study (Supplementary Table S1). The recommended barcoding SNP for L1.2.1 (3479545 C/A), however, was not found.

**Figure 2:**
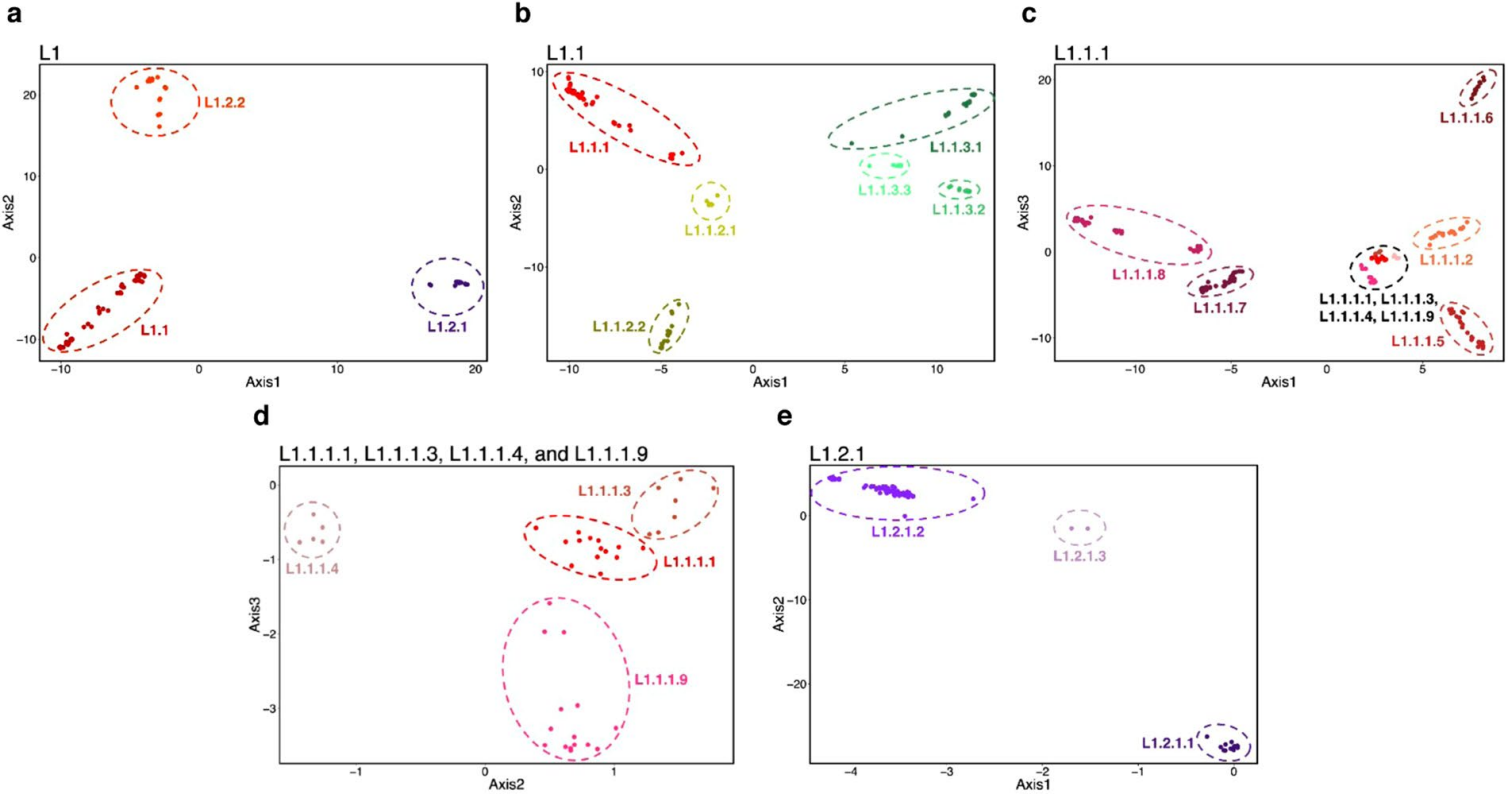
PCA plots revealed the separation of sublineages. (**a**) shows a plot of representative isolates of L1 with axes representing eigenvector 1 and 2. The red, purple and orange dots indicate isolates belonging to L1.1, L1.2.1 and L1.2.2 respectively. (**b**) shows a plot of representative isolates of L1.1 with axes representing eigenvector 1 and 2. Members of L1.1.1 are represented in red. The sublineages of L1.1.2 and L1.1.3 are indicated in different shades of greyish green and green, respectively. (**c**) shows a plot of representative isolates of L1.1.1 with axes representing eigenvector 1 and 3. (**d**) shows a plot of representative isolates of L1.1.1.1, L1.1.1.3, L1.1.1.4 and L1.1.1.9 with axes representing eigenvector 2 and 3. (**e**) shows a plot of representative isolates of L1.2.1 with axes representing eigenvector 1 and 2. L1.2.1.1, L1.2.1.2 and L1.2.1.3 are indicated in dark purple, blue and light purple, respectively.

The topological relationships between the three groups were different. Here, L1.2.2 appeared slightly more related to L1.1, but not L1.2.1, conforming to the presence of isolates with a single copy of IS*6110* only in L1.1 and L1.2.2 but not in L1.2.1 (Supplementary Table S3). The MPSDs between 1.1–1.2.1, 1.1–1.2.2 and 1.2.1–1.2.2 were 800.8, 853.6 and 846.1 respectively, which contributed to the major peak (peak A) in the frequency distribution curve of the pairwise SNV distances, as shown in Supplementary Fig. S2. The nearly equal distances indicated that L1 could be divided into three distinct sublineages, as also shown by the PCA plot in Fig. 2a. The numbers of isolates belonging to the three sublineages were 354 (73.75%) for L1.1, 108 (22.5%) for L1.2.1 and 18 (4.75%) for L1.2.2. Because the number of L1.2.2 isolates was less than in Coll’s report^27^ and all were from only Thailand, the topology of our trees must be confirmed and the name L1.2.2 was maintained throughout this paper.

L1.1 itself comprises three further sublineages, 1.1.1–1.1.3, with all the sublineage-specific SNPs herein previously identified in Coll’s report^27^. The separation was supported by the bootstrap score of 100%, PCA (Fig. 2b) and the fixation indices between 0.33–0.4 (Table 1). The MPSDs between the three groups were approximately 750 (Supplementary Table S4 and Supplementary Fig. S2). The proportions of isolates belonging to these three sublineages were 76.0% (269) for L1.1.1, 9.0% (32) for L1.1.2 and 15.0% (53) for L1.1.3, which differed considerably from Coll’s report. The recommended specific barcoding SNPs for both L1.1.2 and L1.1.3 were not identified herein, suggesting the presence of previously unidentified subgroups in this study. The recommended barcoding SNP for L1.1.2^27^ was specific to a subset, L1.1.2.2, instead, indicating that L1.1.2.1 isolates were not included in Coll’s study.

**Table 1:** Summary of findings for each sublineage in this study.

Sublineages	Number of isolates	Number of specific SNVs	Shared DVR deletions	Coll’s classifi-cation	Number of specific SNPs in Coll’s study	Common specific SNPs in both studies	Confirmed barcoding SNPs	Mean distances within sublineages /between sublineages	Probability	Fixation Indices	Depths/heights in the phylogenetic tree
**1**	480	550		**1**	473		615938 G/A				0/23
**1**.**1**	354	23		**1**.**1**	38	22	4404247 G/A	570.0/808.4.1	<2.2 × 10^−16^	0.295	2/21
**1**.**1**.**1**	269	39		**1**.**1**.**1**	57	39	3021283 G/A	451.1/749.7	<2.2 × 10^−16^	0.398	3/20
**1**.**1**.**1**.**1**	14	44		**1**.**1**.**1**.**1**	138	44	3216553 G/A	252.9/468.3	7.45 × 10^−6^	0.460	8/8
1.1.1.2	26	42						340.5/554.2	6.53 × 10^−10^	0.386	7/10
1.1.1.3	7	5						431.8/503.7	0.002331	0.143*	7/3
1.1.1.4	5	47						224.2/477.2	0.01167*	0.530	11/4
1.1.1.5	42	46	18–21					323.9/505.5	3.12 × 10^−15^	0.359	11/11
1.1.1.6	27	90	57–60					135.3/490.6	3.02 × 10^−10^	0.724	10/11
1.1.1.7	44	29						269.8/453.4	6.79 × 10^−16^	0.405	10/12
1.1.1.8	73	34	53					174.4/467.0	2.2 × 10^−16^	0.627	10/13
1.1.1.9	20	23	62 (not unique)					284.7/487.3	1.45 × 10^−11^	0.416	9/12
Unclassified	11										
**1**.**1**.**2**	32	12		**1**.**1**.**2**	154	12		487.5/750.7	6.49 × 10^−12^	0.351	3/9
1.1.2.1	23	143	18					322.0/720.5	6.60 × 10^−09^	0.553	4/8
1.1.2.2	9	102	3–4				2622402 G/A**	310.0/720.5	4.11 × 10^−5^	0.570	4/5
**1**.**1**.**3 (EAI6)**	53	29	33, 56	**1**.**1**.**3**	66	29	—	502.1/750.2	<2.2 × 10^−16^	0.331	3/11
1.1.3.1	21	85	6–7, 33, 51, 56–57					228.6/654.6	3.12 × 10^−8^	0.651	5/10
1.1.3.2	17	215	33, 56					131.4/671.9	6.99 × 10^−7^	0.805	5/9
1.1.3.3	15	125	33, 56					138.4/652.3	3.38 × 10^−6^	0.788	4/9
**1**.**2**.**1 (EAI2)**	108	208	4, 10	**1**.**2**.**1**	87	55	—	145.3/803.0	<2.2 × 10^−16^	0.819	1/14
1.2.1.1 (EAI2_MNL)	12	63	4,10, 30–31					120.3/286.8	3.64 × 10^−05^	0.580	3/12
1.2.1.2 (EAI2_NTB)	94	20	4,10, 17–35					106.4/272.8	<2.2 × 10^−16^	0.610	2/8
1.2.1.3	2	53	3–4, 10, 30–31					76.0/201.9	0.2207*	0.614	3/1
**1**.**2**.**2**	18	150	62 (not unique)	**1**.**2**.**2**	95	59	3470377 C/T	430.2/851.9	3.22 × 10^−07^	0.495	2/7

The sublineage numbers in bold conforms to the nomenclature of Coll’s *et al*.^27^, with the additional sublineages, named in this study, shown in regular fonts. Specific SNPs appeared in all members of the sublineages. All members of some sublineages shared DVR deletions, but the DVRs may have been deleted sporadically from members of other sublineages. Common specific SNPs in both studies indicates the number of specific SNPs that were the same in both Coll’s and this study. The probability denotes the probability that the mean SNV distances of isolates in a group from the other members in the same group were different from the ones from other isolates that were not in the same group but in the same level of grouping, by the Wilcoxon rank-sum test.

The presence of the other peaks corresponding to smaller distances in the frequency distribution curve of pairwise SNV distances (Supplementary Fig. S2), as well as the large number of isolates, suggested the possibility for further sub-classification. In total, 18 sublineages were tentatively identified, with nine of them in 1.1.1, two in 1.1.2, three each in 1.1.3 and L1.2.1, and one in 1.2.2. The information regarding each sublineage is summarized in Tables 1 and 2. The classification is supported by the phylogenetic trees, with each sublineage having a bootstrap score of 100% and a posterior Bayesian probability of 1.0, and the results of the PCA plots (Fig. 2c–e). This classification is also supported by the statistically significant differences between the π_within group_ and π_between group_ (Wilcoxon rank-sum test with Bonferroni correction, α < 0.00278) or the fixation indices greater than 0.33 (Table 1). Only L1.1.1.4 and L1.2.1.3 had high fixation indices but insignificant differences between π_within group_ and π_between group_, probably due to the low sample numbers. L1.1.1.3 had a low fixation index but statistically significant difference between π_within group_ and π_between group_. The inter-group MPSDs of sublineages of L1.1.1 were mostly around 450–550 (Supplementary Table S4) and contributed to peak B in Supplementary Fig. S2. Eleven isolates were not classified into any groups. Numbering of the sublineages of L1.1.1 was performed so that L1.1.1.1 was the same as in Coll’s assignment^27^, while the others were numbered sequentially according to their positions in the Bayesian tree. The MPSDs between each sublineage are shown in Supplementary Table S4.

**Table 2:** The number of isolates with various experimental spoligotypes identified in each sublineage, listed in ascending order of the octal codes.

Major sublineage	Sub-lineage	Num-ber	Number of isolates × known spoligotypes (SIT)	Number of isolates × unclassified spoligotypes
1.1.1	1.1.1.1	14	1 × 677777777413771 (342), 2 × 777737777413771 (618), **2** × **777777774413771** (**139**), **8** × **777777777413771** (**236**)	1 × 717777777003371
	1.1.1.2	26	**1** **×** **737777777413771** (**204**), 3 × 777737777413731 (349), **1** **×** **777737777413771** (**618**), **2** × **777777777413731** (**48**), **1** **×** **777777777413671** (**256**), **11** × **777777777413771** (**236**), 1 × 777777777773771 (100), 3 × 777777777777771 (523)	1 × 737777770003771, 1 × 777717777413771 1 × 777777777411771*
	1.1.1.3	7	2 × 763777777413771 (792), 1 × 777777767413731 (1404), **3** × **777777777413771** (**236**)	1 × 777777777410071
	1.1.1.4	5	**3** × **777777777413731(48)**, **1** × **777777777413771** (**236**)	1 × 777777767413371
	1.1.1.5	42	15 × 774177777413731 (493), 14 × 77417777741377 (1951), 3 × 774177757413771 orphan	1 × 574177777413731, 1 × 674167777413731, 3 × 774000017413771, 1 × 774077777410071, 1 × 774177777411771, 2 × 774177777413001, 1 × 774177777413701
	1.1.1.6	27	**1** × **777777777413731**๖**48**)*, **26** × **777777777413771** (**236**)	None
	1.1.1.7	44	1 × 777717777413671 (orphan), 1 × 777577777413771(1372), 2 × 777777637413771 (orphan), 1 × 777777760000000 (786), 1 × 777777760000031 (773), 12 × 777777760000071 (1274), 4 × 777777777013771 (937), 1 × 777777777413071 (934), 3 × 777777777413700 (138), **11** × **777777777413771** (**236**)	4 × 703577777413771 1 × 776377777413771 2 × 777777677413700
	1.1.1.8	73	**56** × **777777777413671**(**256**)* (Discrepancy in 3 cases), 1 × 757777777413671 (orphan)*, **2** × **777777777413631** (**947**)	3 × 577777777413671, 1 × 737777777413671, 2 × 777770777413671, 2 × 777777607413671, 1 × 777777777413411, 3 × 777777777413660, 1 × 777777777413661
	1.1.1.9	20	1 × 717777777413731 (1316), 2 × 677777777413731 (529), 1 × 777776777413731 (735), 1 × 777777777413131 (745), **6** × **777777777413631** (**947**), 1 × 777777777413730 (1801), **8** × **777777777413731** (**48**)	1 × 677760377413731
1.1.2	1.1.2.1	23	15 × 775777777413771 (939)	1 × 775000007413771, 1 × 775777757413771*, 1 × 775777777403171, 4 × 775777777413471, 1 × 775777777413761
	1.1.2.2	9	1 × 400037777413771 (8)*, 1 × 474000377413031 (1983), 1 × 475777777413771 (Orphan)*, 1 × 477001777413771 (1875), 1 × 477777777413031 (355), 2 × 477777777413071 (11), 1 × 477777777413731 (1182)*	1 × 475777777413051*
1.1.3 (EAI6_BGD1)	1.1.3.1	21	13 × 777777757413371 (292), 1 × 777777700003371 (1391), 2 × 777777743413371 (Orphan), 1 × 777777747413371 (1390)	1 × 777577757413371 1 × 777767700003371* 1 × 777777707413371 1 × 777777750403371
	1.1.3.2	17	**12** × **777777757413771** (**591**), 2 × 737777757413771 (orphan)	3 × 777777657413771
	1.1.3.3	15	**11** × **777777757413771** (**591**)	1 × 777701757413771, 1 × 777777703413771, 2 × 777777757412771*
1.2.1 (EAI2)	1.2.1.1 (EAI2_MNL)	12	1 × 677767477413771 (1490), 1 × 677777477413701 (483), 1 × 677777477413751 (287), 9 × 677777477413771 (19)	None
	1.2.1.2 (EAI2_NTB)	94	83 × 674000003413771 (89)* (Discrepancy in 6 cases) 1 × 074000003413771 (orphan), 1 × 600000000000000 (orphan), 4 × 674000002000071 (orphan)	1 × 474000003413771, 2 × 674000003413700, 2 × 674000003413711
	1.2.1.3	2	1 × 477777477413771 (413)	1 × 477777477413731
1.2.2	1.2.2	18	2 × 577777777413731 (477), 1 × 777767777413731 (1251)*, 2 × 777777774413731 (514), **1** × **777777777413631** (**947**), **1** × **777777777413711** (**517**), 6 × 777777777413731 (48)	1 × 776177775413731, 1 × 777777774403731*, 1 × 777777774412731, 1 × 777777775413731, 1 × 777777777411631
	Un-classified	11	**2** × **737777777413771** (**204**), 3 × 777777774000071 (944), **1** × **777777757413771** (**591**), **2** × **777777777413711** (**517**), **1** × **777777777413731** (**48**)	1 × 773637777413771, 1 × 777637777413411

The spoligotypes that are found in more than one sublineage are indicated in bold typeface. No unclassified spoligotypes appeared in two sublineages. (SIT denotes the spoligotype international type).

*The spoligotypes, of which some isolates had different predicted spoligotypes.

This classification should provide a framework for identifying more variants belonging to L1 as well as for comparison of WGS across countries. The SNPs specific to each sublineage are provided in Supplementary Table S1. It is conceivable that the specific SNPs for each sublineage may decrease when more samples are examined.

As indicated by the intragroup MPSD, the diversity within each sublineage varied, which may be related to the level of transmission in Chiangrai. A smaller MPSD indicates a closer relationship between members of the sublineage, which may result from higher transmission activity, either in Chiangrai or in the vicinity. A large MPSD indicates that most isolates in the sublineage are distantly related and might have been introduced into Chiangrai separately. For L1.1.3, both events might occur. The MPSD of L1.1.3 was approximately three times higher than that of its three sublineages, suggesting that there were at least three clades belonging to L1.1.3, each of which was probably separately introduced to Chiangrai.

### Concordance between the SNV phylogenetic tree and clades defined by spoligotypes

The identification of DR regions from the DNA contigs was successful in 476 of 480 isolates. Unfortunately, the identification of DR regions in the non-EAI isolates, belonging to SIT100 and SIT513, was unsuccessful. A comparison of the identified DVRs with experimental spoligotypes revealed 23 cases (4.8%) of discrepancy. In seven cases, several DVRs were not identified computationally but they were detected by experimental spoligotyping, shown in Table 2. The discrepancy in the other cases was only one or two DVRs.

The SNV phylogeny indicated that EAI2_MNL, EAI2_NTB and EAI6_BGD1 were monophyletic clades, corresponding exactly to L1.2.1.1, L1.2.1.2, and L1.1.3 respectively. Their DR regions were characterized by multiple deletions compared with the EAI_MRCA (Table 1). The multiple deletion events should result in low probability of homoplasy and indicate common ancestors.

Comparison between SNV phylogeny and spoligotypes confirmed the notion that the DR region evolved mostly by deletions. All L1.1.3 isolates shared the deletions of DVR33 and 56, while L1.1.3.1 had additional deletions of DVR6-7, 51 and 57. All L1.2.1 isolates shared the deletions of DVR4 and 10. Each of its three sublineages, L1.2.1.1, L1.2.1.2 and L1.2.1.3 had additional deletions of DVR30-31, DVR17-35 and DVR3 respectively.

An experimental EAI6_BGD1 isolate did not belong to L1.1.3. In fact, the isolate still had the unspoligotyped DVR56. Extending the spoligotyping to cover the entire DR region would easily differentiate it from L1.1.3.

### Lack of correspondence between SNV phylogenetic trees and some spoligotypic clades

There was insufficient information to confirm that EAI3 was monophyletic but isolates classified as EAI4_VNM were found in both L1.2.2 and L1.1.1.1, suggesting that EAI4_VNM might not be monophyletic. It was clear, however, that EAI1 and EAI5 were not monophyletic. Both belonged to many sublineages and appeared together in some sublineages.

There were 60 isolates, with SIT236 (777777777413771), typical for EAI5, belonging to 6 sublineages of L1.1.1 (Table 2). The isolates contained all spoligotyped DVRs, except DVR39-42 and 44. Thus, SIT236 was not defined by any specific deletion but instead by the lack of any deletion in the spoligotyped DVRs. Twenty-one isolates (34%) contained all 62 DVRs apart from the six EAI-defining DVRs; therefore, they should have the DR region similar to the EAI-MRCA. This result indicated that some descendants of EAI-MRCA had maintained the DR region without further deletions. Nevertheless, they separately accumulated different mutations and became different sublineages of L1.1.1; specifically, L1.1.1.1- L1.1.1.4 and L1.1.1.7.

A similar situation was observed with EAI1. The common EAI1 spoligotype, SIT48 (777777777413731) has only one more deleted DVR, DVR62, compared with SIT236. In this study, 14 of 20 isolates with SIT48, belonging to L1.2.2, L1.1.1.4 and L1.1.1.9, retained all the other unspoligotyped DVRs. As L1.2.2 separated from the other sublineages early in the evolution of L1 (Fig. 1), its DVR62 deletion must also occur very early. However, because DVR62-deleted isolates were also observed in many sublineages of L1.1, sometimes in the same sublineage as isolates with the intact DVR62, the DVR62 deletions in L1.1 must be separate, homoplastic, events. Isolates with SIT48 and SIT236 have been reported in wide geographic areas (Supplementary Table S7). Still, it cannot be concluded that the isolates belonged to the same sublineages.

There were some sublineages associated with single DVR deletions such as L1.1.2.1-DVR18, L1.1.1.5-DVR18-21, L1.1.1.8-DVR53, and L1.1.1.6-DVR57-60. However, the associations were not completely specific and the single deletion event might allow for homoplasy, as in the case of DVR62 deletion.

### Limited association between SNP phylogeny and IS*6110* copy number

*M. tuberculosis* isolates from South and Southeast Asia usually have a single copy or low copy numbers of IS*6110*^32^. Our study revealed that the isolates could belong to many sublineages except for L1.2.1 and L1.1.3 (Supplementary Table S3). Unless the DVR34 and DVR35 were deleted, the single copy was always found between them.

### No association of patient profiles with sublineages except for age

The profiles of the patients infected by each major sublineage are shown in Table 3, of whom 89% were Thais, 3% were foreigners and the remaining 8% belonged to one of the several hill tribes. The majority of patients (73.8%) were male with a median age of 50 years. The proportions of patients over age 49 infected by various sublineages were significantly different (Chi-square test, p = 0.0246). The relative risk of patients over age 49 being infected by L1.1.1 compared to the patients with younger age was 1.29 (p = 0.0019, CI:1.099-1.522). There was no association between HIV infection and the sublineages.

**Table 3:** Demographic and clinical profiles of patients infected by different sublineages in this study.

	L1.1.1	L1.1.2	L1.1.3	L1.2.1	L1.2.2	All
Total	269	32	53	108	18	480
**Sex**						
Male	198	27	37	78	14	354
Female	71	5	16	30	4	126
**Ages**						
<19	1	0	2	4	0	7
20–29	24	2	4	12	1	43
30–39	42	6	15	25	4	92
40–49	47	8	12	20	5	92
50–59	57	5	7	18	1	88
60–69	50	9	10	11	3	83
>70	48	2	3	18	4	75
Average ages	53.2	50.3	45.6	48.5	51.9	50.7
Median ages	54	49	43	46	47	50
% with ages >49*	57.6	50.0	37.7	43.5	44.4	51.3
**Ethnicity**						
Thai	238	26	45	103	17	429
Hill tribe	24	3	6	3	1	37
Foreigner	7	3	2	2	0	14
**Clinical presentation**						
Pulmonary	258	32	49	102	17	458
Pulmonary and Extrapulmonary	9	0	4	6	1	20
Extrapulmonary	2	0	0	0	0	2
**HIV status**						
Positive	49	5	12	29	4	99
Negative	215	27	40	76	13	371
Unknown	5	0	1	3	1	10

*The proportions of the patients over age 49 were different between various sublineages (Chi-square test, p = 0.0246). The relative risk of infecting by L1.1.1 of patients over age 49, compared to the ones being younger was 1.29 (p = 0.0019, 95% CI: 1.099–1.522).

### Similarity of population structures between various countries

As the informations regarding the SNP-based genetic population structures of L1 in various endemic countries are very limited while the informations on spoligotypes are widely available, we investigated the similarity of population structures of L1 by examining the correlation of spoligotype profiles and the similarity of the most predominant clades. The SNV phylogeny above allowed the exclusion of the homoplastic spoligotypes, which belonged to many sublineages, such as SIT48 and SIT236.

### Correlation between spoligotypes in various countries

The correlation of frequencies of EAI spoligotypes between various studies was calculated by excluding the data for homoplastic spoligotypes. Although each study had different and sometimes unspecified sampling designs, which mandates caution in interpreting the results, several general patterns can be observed (Supplementary Table S8). There were no correlations of spoligotype profiles between countries in East Africa (excluding Madagascar) and Southeast Asia.

Strong correlations (>0.8) were usually found between studies performed in the same country, as expected, which might not be true if a large number of samples were intentionally selected from patients with specific forms of the disease, such as extrapulmonary or drug-resistant tuberculosis. There were numerous studies in India, where the percentages of L1 are known to be high in the southern and low in the northern part of the country. Nevertheless, the L1 spoligotype profiles were generally similar throughout the country, excluding eastern India.

Correlations between studies in bordering countries were usually intermediate and higher than correlations between countries without common borders. Strong correlations were identified between Pakistan and Indian studies, particularly when homoplastic spoligotypes were excluded, conforming to the close historical relationship between the two countries. There were also strong correlations between the spoligotype profiles in Bangladesh and Myanmar (Supplementary Table S8).

Intriguing patterns of spoligotype profile correlations were observed in Southeast Asian countries. Countries in islands of Southeast Asia (ISEA), Indonesia and the Philippines, as well as Singapore and Taiwan are separated by seas but share similar L1 spoligotype profiles comprising predominantly EAI2_MNL (L1.2.1.1). In contrast, there was low similarity of spoligotype profiles between MSEA countries. In all cases, there were some levels of similarity of spoligotype profiles only between countries that shared common borders. The correlation coefficients between spoligotype profiles between countries without common borders were always low (Supplementary Table S8).

### Literature review indicating that the predominant sublineages in each country were usually different

**Figure 3:**
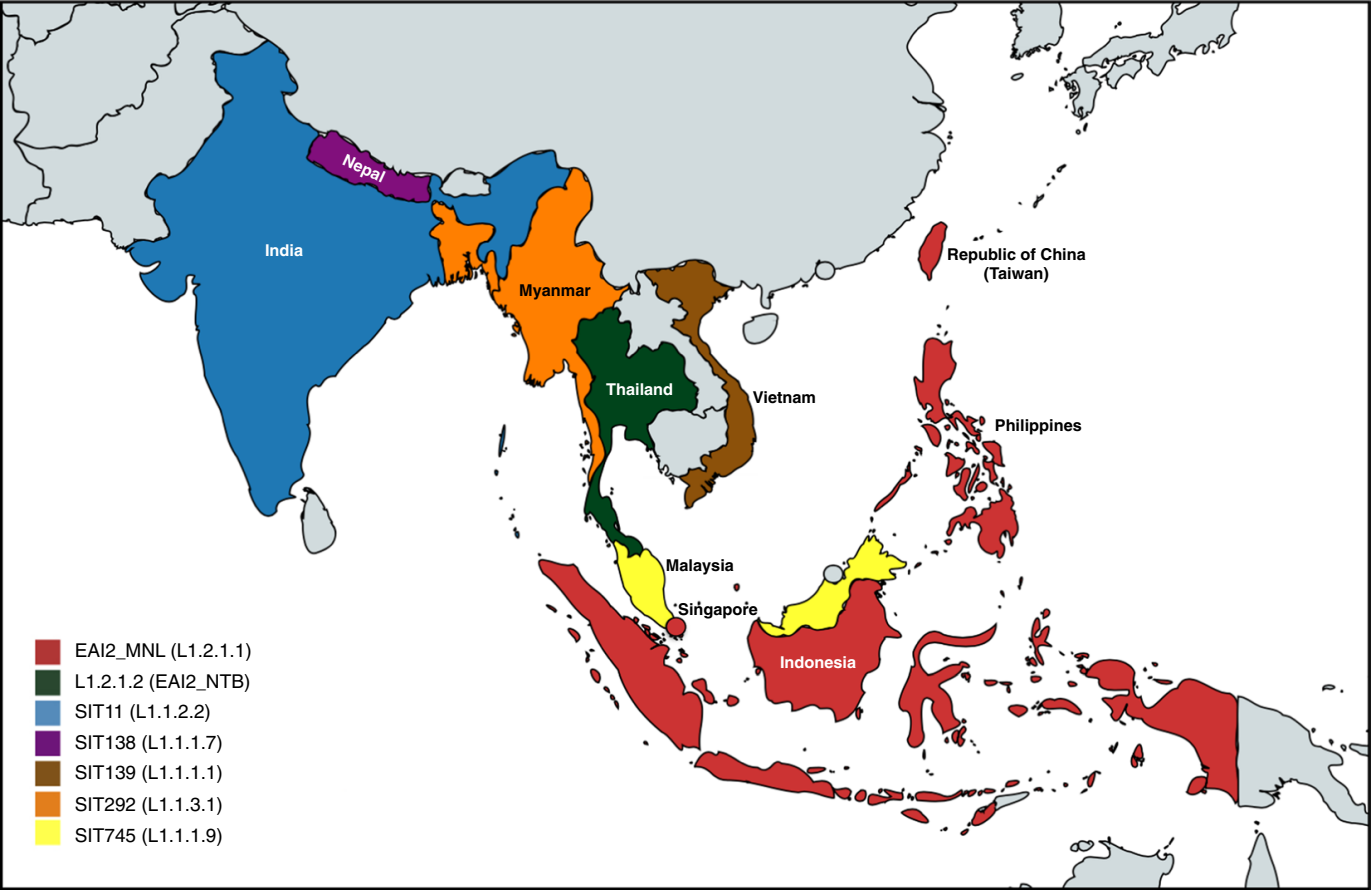
Area map showing the predominant sublineages of L1 in different countries in Southeast Asia, deduced from their spoligotype reports. There were no obvious predominant strains in Cambodia and there was no information from Lao.

The reported predominant spoligotypes in various countries were usually different, as shown in Fig. 3 and Supplementary Table S7. Countries that had similar profiles usually shared the same predominant spoligotypes.

### Predominant sublineages in Chiangrai were not reported frequently in neighbouring countries

The two most common sublineages (L1.2.1.2 and L1.1.1.8) contributed to a third of L1 isolates in Chiangrai. Of the 94 members of L1.2.1.2, 83 (88%) had SIT89 or EAI2_NTB while the remaining 11 had new spoligotypes that could be derived from SIT89 by additional DVR deletions. The family was initially recognized by IS*6110*-RFLP in Nonthaburi, central Thailand^32^. All 73 L1.1.1.8 isolates had a characteristic deletion of DVR53, with 56 (77%) isolates having SIT256, while the others had spoligotypes that could be derived from SIT256 by deletion. Both L1.2.1.2 and L1.1.1.8 had very low intragroup MPSDs of 106.4 and 174.4 and very high fixation indices of 0.61 and 0.63 respectively, indicating a close genetic relationship within each group. Together with the high prevalence, these results suggested high transmission activities of both sublineages.

In countries bordering Thailand, Cambodia, Malaysia and Myanmar, both SIT89 and SIT256 were reported but at much lower frequencies (Supplementary Table S7). This result suggested that their transmission activities were greater in Chiangrai. It can, therefore, be hypothesized that L1.2.1.2 and L1.1.1.8 might be better adapted to the populations in Chiangrai.

SIT292, the most common spoligotype in L1.1.3.1, was predominant in Myanmar and Bangladesh and also found in many patients in Chiangrai. People from both countries contributed substantially to the workforce population in Chiangrai (Supplementary Table S7).

## Discussion

In this study, we analysed WGS of 480 L1 isolates and refined its classification. The results should facilitate comparison of WGS information across countries. Comparisons of phylogenetic trees with spoligotypes indicated the benefits of using all 68 DVRs instead of only 43 for spoligotyping.

The availability of spoligotype information in many countries indicated that the predominant sublineages in each country varied with geography and apparently with ethnicity.

EAI2_MNL or L1.2.1.1 was highly prevalent in the Philippines, accounting for more than 80% of all *M. tuberculosis* isolates, and in Southern Taiwan and Indonesia, where it accounted for >90% and approximately 40% of EAI isolates respectively (Supplementary Tables S6 and S7). ISEA, including the Philippines and Indonesia, are home to people who speak Austronesian languages that originated in Southern Taiwan approximately 5000 years ago and spread throughout the Pacific islands and to Madagascar^33,34^. A few spoligotyping studies in other island countries, such as in Kiribati^35^ and Papua New Guinea^36,37^, revealed the rarity of the EAI isolates. The rare EAI isolates, however, usually belonged to EAI2_MNL, which was also reported from Hawaii and Guam^38^. Thus, L1.2.1.1 may associate with Austronesians either due to the founder effect or co-evolution. Furthermore, the immediate prediction is that the common spoligotype in Madagascar, SIT109 (EAI8_MDG)^39^, should also belong to L1.2.1.1.

In contrast to ISEA, countries in MSEA are geographically closer but populated by ethnic groups that speak different language families, suggesting different genetic roots. Most Vietnamese people belong to the Kinh ethnic group and speak the Vietic branch of Austroasiatic language^40^, while Cambodians speak the Khmer branch. The Thai language belongs to the Tai-Kadai family while Burmese belongs to the Tibeto-Burman family. The Malay language belongs to the Malayo-Polynesian branch of the Austronesian family. Each country also had a different predominant spoligotype belonging to different sublineages (Fig. 3), while there was no clear predominant spoligotype in Cambodia (Supplementary Table S7).

The differences in the predominant L1 strains and spoligotype profiles in MSEA countries are intriguing because they have a history of extensive interactions accompanied by alternative expansion and contraction of territorial control for over a millennium. Nevertheless, the inter-country variations of *M. tuberculosis* were still obvious, suggesting a contribution of co-adaptation between the bacteria and theirhuman hosts. The results herein, therefore, provided supportive evidence for the co-adaptation hypothesis in the case of L1, similar to the finding in lineage 4^7^. Due to the poor uniformity of each spoligotyping study, as shown in supplementary Table S6, rigorous confirmation of the hypothesis will require WGS of representative samples of bacterial populations from many countries around the Indian Ocean.

The predominant L1 spoligotype in Malaysia was SIT745 (27%), while EAI2_MNL accounted for approximately 9%^16^. Although the native language of many Malaysians is Austronesian, similar to people in ISEA, current Malaysians may be a genetic admixture between people who were originally in MSEA and ISEA^41^.

The most common spoligotype in Vietnam was uniquely SIT139, a rare spoligotype classified as L1.1.1.1 in this study, accounting for 48% of Vietnamese L1 isolates^17^, which might indicate a specific adaptation. In contrast, L1.2.1.1 was rare in Vietnam. This result is intriguing because the Cham people in the historical Champa Kingdom, in what is now southern Vietnam, spoke Austronesian. The kingdom lasted for several centuries and afforded ample interactions between Cham and Kinh, the major ethnic group in Vietnam. The rarity of L1.2.1.1 could be a consequence of the Champa Kingdom acquiring mainly Austronesian languages and culture rather than a large migration of genetic Austronesians^42^.

Existing studies on the transmissibility of L1 supported the co-evolution hypothesis. A population-based study in Southern Taiwan revealed that the genotypic clustering rates of 60.8% among EAI strains^43^, which mostly belonged to L1.2.1.1, suggesting high transmissibility. Another study in Vietnam reported the clustering rate among EAI2_MNL (L1.2.1.1), an uncommon sublineage in that region, of only 18.3% while the clustering rate among EAI4_VNM, the predominant clade including SIT139, was 77.4%^44^. This result suggested that L1.2.1.1 and SIT139 spread differently in both populations, conforming to the co-adaptation hypothesis.

Robust phylogenetics provides a foundation for genotype-phenotype correlation studies. In general, it is difficult to compare phenotypes of *M. tuberculosis* lineages at the population level across several studies. Although many recent studies provided genotypic details of the studied isolates, their phylogenetic relationships were usually not considered in the analysis^45^. Moreover, many studies used the phenotypes of the other strains in the same studied areas as the controls. The controls, therefore, varied between studies in both proportions of lineages and sublineages. Moreover, in most studies, information regarding host population genetic structures are usually not available.

*In vitro* phenotypic studies of *M. tuberculosis* typically involved a small number of strains and rarely had sublineage information, rendering comparison between studies difficult. A few recent studies, involving L1 isolates with sublineage information, included the autophagy study of EAI2_NTB (L1.2.1.2)^46^. A number of EAI isolates belonging to SIT1390 (L1.1.3.1)^23^ or EAI2_MNL (L1.2.1.1)^22,23,47^ were shown to induce high levels of inflammatory cytokines. Unfortunately, there are no known *in vitro* studies involving L1.1.1 or L1.1.2 isolates that merit further exploration.

India harbours a quarter of the world patient population^1^, its predominant strain, SIT11 belonging to L1.1.2.2 (Supplementary Table S7), may be one of the genotypes infecting the highest absolute number of people, similar to L1.2.1.1, which is common in ISEA^1^. Recently, it was found that 90% of *M. tuberculosis* in Tamil Nadu, Southern India, where the Chingleput BCG trials had been conducted^26^, were either EAI3, including SIT11, or EAI5^48^, which should belong to L1.1.2 or L1.1.1 respectively. Further genomics and pathogenesis studies of the sublineages may provide important clues for the development of new tuberculosis vaccines.

The co-adaptation between *M. tuberculosis* and its hosts may explain the difficulty in consistently identifying human genetic markers associated with tuberculosis^49^. Considering that the host genetic susceptibility risks for tuberculosis are likely oligogenic and may not be similar across multiple populations with large genetic distances, it is possible that host susceptibility follows a genetic heterogeneity model, in which a group of people with specific genetic susceptibility allows better local spreading of some sublineages of *M. tuberculosis*^50^. The susceptibility may be mild and significant only among those who have high genetic similarity, such as family members, which may result in a higher prevalence in a genetically related population. Consequently considering the genotypic profiles of *M. tuberculosis* as strata of differential susceptibility risk factors in host susceptibility analysis may result in the successful identification of the host genetic factors^9,10^.

In conclusion, 18 tentative L1 sublineages belonging to three major groups were identified. The limitations of standard spoligotyping were revealed, favouring extended spoligotyping methods using all 68 DVRs. The phylogenetic trees suggested relatively high transmission activities in some sublineages, inferring possible selective advantages. However, some common spoligotypes in neighbouring countries were uncommon in Chiangrai, suggesting different levels of transmission in different countries. This conforms to the notion that *M. tuberculosis* sublineage adapt to the host populations. This finding has implications for studies on the host-pathogen relationship such as GWAS, and may have implications in the urgent need for vaccine testing and development.

## Materials and Methods

### Settings

This study included *M. tuberculosis* isolates from patients in Chiangrai Province, Northern Thailand from 2003–2010. Chiangrai borders both Myanmar and Lao and is an important transportation hub with China through the Mekong River. The population of the province consists of approximately 1.2 million people with a tuberculosis incidence rate of 152.6/100,000 population in 2011.

The isolates were collected as part of a tuberculosis cohort study initiated by the Japan Research Institute of Tuberculosis (RIT), the Japan Anti-Tuberculosis Association (JATA), and the Ministry of Public Health, Thailand. The study recruited all newly-diagnosed culture positive tuberculosis patients with all nationality who were also willing to participate in a human genetic study from all pubic hospitals in Chiangrai. During the studied period, there were 15,805 new cases in Public Hospital Tuberculosis Registration System. Bacterial isolates were obtained from 7148 patients, of which both bacterial and host DNA were obtained from 1187 patients. 1174 M. tuberculosis isolates were successfully sequenced and 480 were identified to be Indo-Oceanic strains.

### Ethics Statement

The project was approved by the Ethical Committees of Chiangrai Prachanukroh Hospital, Chiangrai and the Thai Ministry of Public Health. Informed consent was obtained from all participants and/or their legal guardians. All methods were performed in accordance with the relevant guidelines and regulations.

### Bacteria

Bacterial samples from 1187 patients were successfully regrown in Lowenstein-Jensen medium in an appropriately contained clinical microbiology laboratory in Chiangrai using standard biosafety protocols and equipment. The bacteria were heat-killed and DNA was prepared as previously described^32^. All the processes were performed in Class II biosafety cabinets.

### Bacterial genotyping

#### Large sequence polymorphisms (LSP)

Lineages of the isolates in this study were initially identified by LSP using PCR primers specific to TbD1 (for differentiation between ancestral and modern strains), RD239 (for lineage 1), RD105 (for lineage 2), RD750 (for lineage 3) and 7 bp sequence at pks15/1 (for lineage 4)^51^. The primer sequences are shown in Supplementary Table S9.

The 487 isolates were identified as Indo-Oceanic strains due the presence of the TbD1 and the absence of RD239.

#### Spoligotyping

Spoligotyping was performed using a commercial kit (Ocimum Biosolutions, India) as previously described^52^. Spoligotyping investigates the presence or absence of 43 of the 68 DVRs. The results were coded into a binary pattern of 43 digits, which is typically converted to an octal code by the successive combination of three binary digits from the left, leaving the 15^th^ code as still binary. *M. tuberculosis* L1 typically has EAI spoligotypes, i.e. the 10–12th octal code of 413. Some octal codes have been designated as spoligotype international type (SIT) and assigned to one of the various clades, such as EAI1, EAI2_MNL, and EAI2_NTB, among others. The SIT and clade assignment was performed by querying SITVITWEB (http://www.pasteur-guadeloupe.fr:8081/SITVIT_ONLINE/; updated February 17, 2017)^30^. Spoligotypes were also predicted from whole-genome sequences using *SpolPred* software^53^.

#### Whole-genome sequencing and SNV analysis

*M. tuberculosis* samples were sequenced on the Illumina HiSeq. 2000 platform at the Wellcome Trust Sanger Institute, UK. The system produced paired-end reads in FastQ file format. We used Trimmomatic version 0.36^54^ to collect only the paired reads. Five isolates were not further analysed because the numbers of available reads were too small. The raw reads were filtered and discarded if their phred quality scores were lower than 20. The remaining reads were aligned by the BWA program^55^ and SAMtools^56^ using *M. tuberculosis* H37Rv (GenBank NC_000962.3) as the reference genome with alignment score more than 50, SAMtools base quality more than 23 and read depth more than 10. Two samples appeared to contain mixed nucleotide sequences and were also not further studied. The SNVs that were present in any drug-resistance gene, mobile genetic element, phage, PE/PPE region and non-homozygous SNVs were discarded. The remaining SNVs had been converted to an SNV-supermatrix using an in-house Python script before being used in the phylogenetic analysis. The sequencing data for the 480 samples used in this paper were submitted to the European Nucleotide Archive (ENA) of EMBL-EBI mirrored in the Sequence Read Archive (SRA) database. Actual read sequences can be queried and downloaded directly from the SRA database using the accession numbers listed in Supplementary Table S10.

#### Phylogenetics analysis

SNVs of 480 Indo-Oceanic isolates were used for phylogenetic tree construction, with 24 isolates from the lineage 2–4 as outgroups. We analysed the generated supermatrix with two phylogenetic analytic methods, maximum likelihood (ML) and Bayesian inference (BI).

For ML analysis, we used PhyML^57^ via the SeaView program^58^. The core tree (starting tree) was generated using the BioNJ method. The analysis was run using GTR model, using the nearest neighbor interchange (NNI) method. The bootstrap analysis was calculated for branch support with 1,000 replications of pseudo-data.

For the BI methods, we used the MrBayes program^54^. The parameter “4by4” was used as the nucleotide substitution model. The BI analysis was performed with two simultaneous runs with four n-chain for each run. The analysis was run for approximately 10–20 million generations. The analysis was terminated when the average standard deviation of split frequencies was lower than 0.01. The first 25% of sample trees were discarded as burned-in. All trees were visualized using the FigTree program version 1.4.2. (http://tree.bio.ed.ac.uk/software/figtree/).

Principal Component Analysis was performed using Jalview 2.8.2^57^. The analysis was conducted at multiple levels of sublineage classification. The plots between the first three eigenvectors were examined.

Pairwise SNV distances were calculated using MEGA5^56^. The fixation index (F_ST_) was calculated based on the following formula (π_between group_ − π_within group_)/π_between group_, where π_within group_ is the average pairwise SNV distance within a group and π_between group_ is the average pairwise SNV distance between all members in the group and all members not in the same group but in the same level of grouping. The statistical tests for the difference between the within-group averages and between-group averages were performed using Wilcoxon rank-sum test at α = 0.05 with Bonferroni correction multiple testing using R statistical package version 3.3.2.

#### Analysis of the Direct Repeat (DR) region and IS*6110* copy number

The sequence contigs of each isolate were constructed *de novo* using velvet software^59^. Analysis of the DR region in each genome was performed by carrying out a blastn search of the contig files containing the DR region. The identified contigs were mapped to areas around the position of 3119181–3123573 in the genome of H37Rv (GenBank NC_000962.3). The presence or absence of each of the 68 known direct variable region (DVR) sequences was determined and recorded as shown in Supplementary Table S5.

The number of IS*6110* in the genome of each isolate was identified by ISMapper using the provided IS*6110* sequence^60^ as the query and H37Rv as the reference genome with default parameters.

#### Analysis of phenotypic data

The patient characteristics were described in descriptive statistics (as presented in Table 3). The association between phenotypes and the five major sublineages of L1 were evaluated by one-tailed Pearson Chi-square test. The P-values less than 0.05 were considered significant. Association with ages were analysed by categorizing patients into two groups, with the age over 49 and 49 or less. The relative risk of patients over age 49 for being infected by L1.1.1 strains was calculated compared to the younger patients.

#### Comparison of spoligotypes between countries

Relevant research articles were identified by PubMed search using the keywords, spoligotype or genotype and tuberculosis and names of the countries in the ASEAN, South Asian and Pacific islands. The full papers were retrieved and examined for the presence of detailed descriptions of spoligotypes. Forty-one articles were identified with 6 having less than 20 EAI isolates in each study. The latter group was excluded. The list of retrieved papers is shown in Supplementary Table S6. The numbers of EAI isolates and isolates belonging to each reported SIT were tabulated, as shown in Supplementary Table S7, with the frequency of each spoligotype calculated. The data for spoligotypes that could appear in more than one sublineages, SIT48, 204, 236, 517, 618 and 947, were excluded. Then pairwise correlations between the frequencies were evaluated by calculating Pearson correlation coefficients. A higher correlation coefficient indicated a greater similarity of the spoligotype profiles between two studies.

## Electronic supplementary material

Supplementary Information

Dataset for supplementary table S1

Dataset for supplementary table S5

Dataset for supplementary table S10
