# Supplementary material for: Evidence for Host-Bacterial Co-evolution via Genome Sequence Analysis of 480 Thai Mycobacterium tuberculosis Lineage 1 Isolates
Source: Sci Rep. 2018 Aug 2;8:11597. doi: 10.1038/s41598-018-29986-3 (PMC6072702; doi:10.1038/s41598-018-29986-3)
Supplement: Supplementary file 1 — Supplementary Information [file 41598_2018_29986_MOESM1_ESM.pdf]

**Supplementary Information for the manuscript**

**Evidence for Host-Bacterial Co-evolution via Genome Sequence Analysis of 480 Thai *Mycobacterium tuberculosis* Lineage 1 Isolates**

Prasit Palittapongarnpim[1 2 *], Pravech Ajawatanawong[1], Wasna Viratyosin[2], Nat Smittipat[2], Areeya Disratthakit[3], Surakameth Mahasirimongkol[3], Hideki Yanai[4 5], Norio Yamada[6], Supalert Nedsuwan[7], Worarat Imasanguan[7], Pacharee Kantipong[7], Boonchai Chaiyasirinroje[4], Jiraporn Wongyai[4], Licht Toyo-oka[8], Jody Phelan[9], Julian Parkhill[10], Taane G. Clark[9], Martin L. Hibberd[9], Wuthiwat Ruengchai[1], Panawun Palittapongarnpim[1], Tada Juthayothin[2], Sissades Tongsima[2], Katsushi Tokunaga[8]

This supplementary PDF file includes the following information:

Supplementary Figures S1-2

Supplementary Table S1-10

References to supplementary information

Note: Supplementary Tables S1, S5, S10 are provided as individual excel files

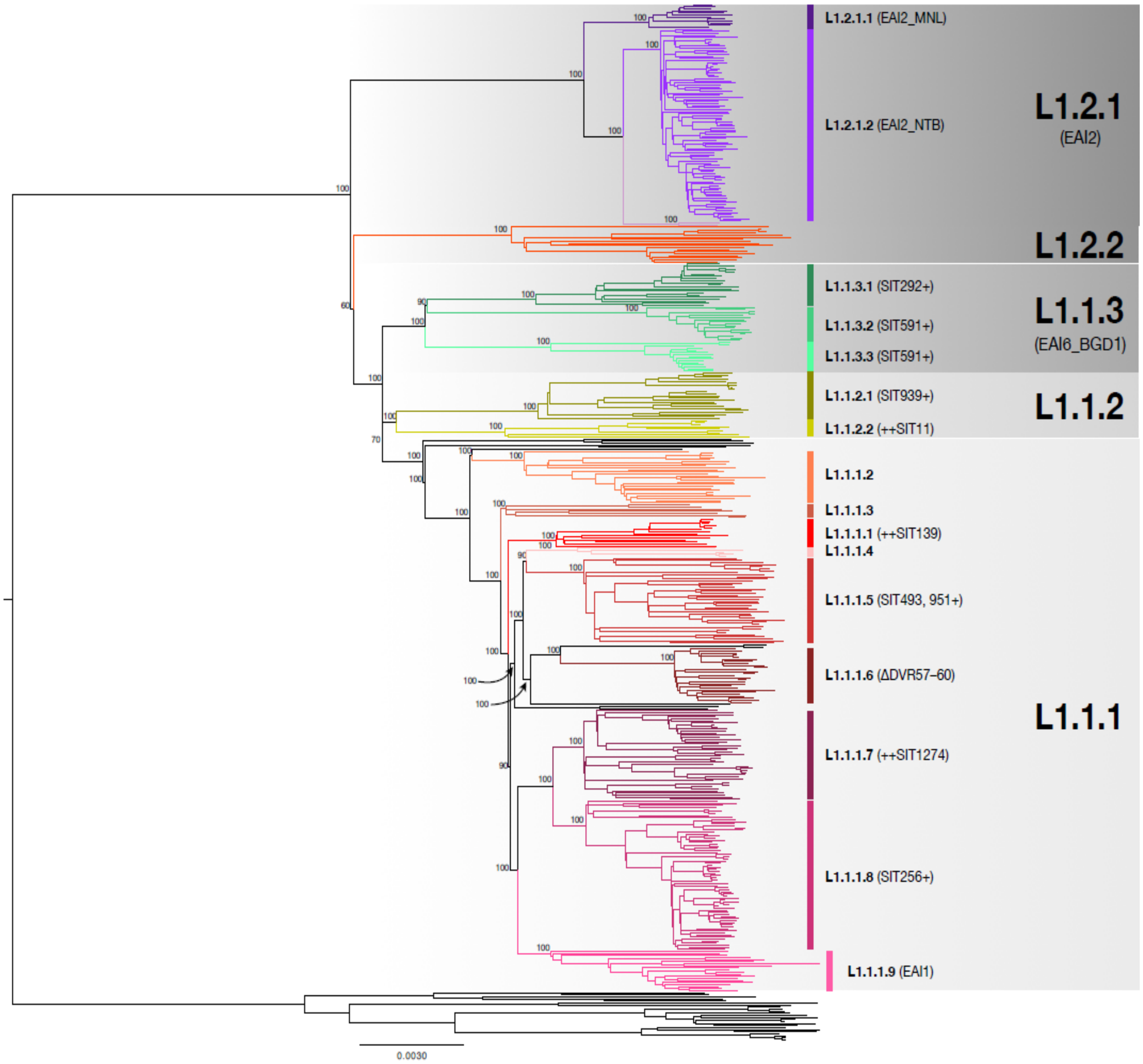


**Supplementary Figure S1.** The phylogenetic tree of 480 *M. tuberculosis* L1 isolates from Chiangrai Province, constructed by the maximum likelihood method.

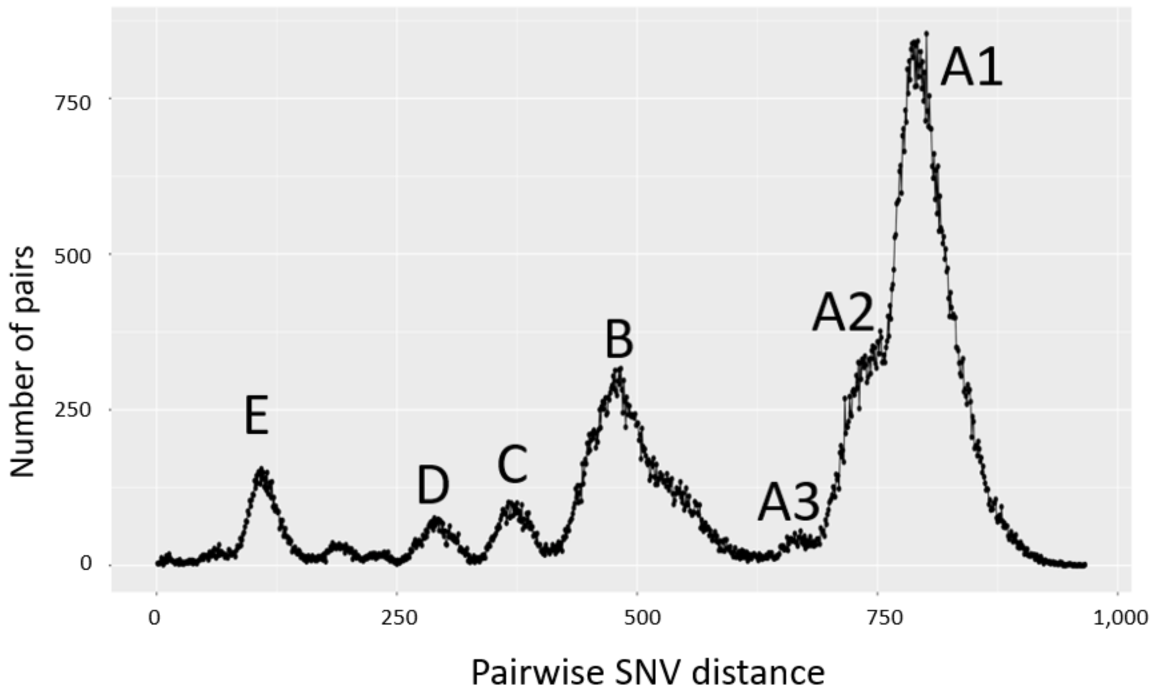


**Supplementary Figure S2**. Numbers of bacterial isolate pairs with different pairwise SNV distances among the 480 L1 isolates. Peak A is contributed mostly by the between-group distances of 3 major groups (L1.1, L1.2.1 and L1.2.2). The shoulder of peak A (A2) is contributed mostly by the between-group distances of 3 major subgroups of L1.1 (L1.1.1-L1.1.3), while the small peak at the base of peak A (A3) is contributed mostly by the between-group distances of 3 sublineages of L1.1.3. Peak B is contributed mostly by the between-group distances of sublineages of L1.1.1. Peak E is contributed mostly by the intragroup distances of L1.2.1.2, L1.1.1.8 and L1.1.1.6.

**Supplementary Table S1.** Specific SNPs for each sublineage in this study are listed in a separate sheet in the excel file: **Palittapongarnpim Final TS1 EAI1_SNPs_list.xlsx**. The specific SNPs displayed in green are the same as in Coll's report[1].

**Supplementary Table S2** Number of SNVs identified among L1 isolates in various studies.

| Reports | Number of samples of L1 | Location | Number of SNVs | Average pairwise SNV distance/ total SNVs |
|---|---|---|---|---|
| Coll, et al. 2014[1] | 121 | Global | 14661 | 0.0102 |
| Comas, et al. 2013[2] | 44 | Global | 15671 | |
| Manson, et al. 2017[3] | 141 | India (two cities) | 24266 | |
| This study | 480 | Thailand (Chiangrai) | 41157 | 0.0157 |

**Supplementary Table S3** Frequency of isolates with different numbers of copies of IS*6110* in each sublineage, determined by ISMapper[4].

| **SNP TYPE** | **0** | **1** | **2** | **3** | **4** | **5** | **6** | **7** | **8** | **9** | **10** | **11** | **12** | **13** | **TOTAL** |
|---|---|---|---|---|---|---|---|---|---|---|---|---|---|---|---|
| **1.1.1** | | | | | | | | | | | | | | | |
| 1.1.1.1 | | 12 | | 1 | 1 | | | | | | | | | | 14 |
| 1.1.1.2 | 2 | 8 | 1 | 6 | 5 | 4 | | | | | | | | | 26 |
| 1.1.1.3 | | 6 | 1 | | | | | | | | | | | | 7 |
| 1.1.1.4 | | 4 | | | | 1 | | | | | | | | | 5 |
| 1.1.1.5 | | 40 | | 1 | 1 | | | | | | | | | | 42 |
| 1.1.1.6 | | 1 | 15 | 9 | 2 | | | | | | | | | | 27 |
| 1.1.1.7 | | 2 | 8 | 17 | 8 | 7 | 1 | 1 | | | | | | | 44 |
| 1.1.1.8 | 1 | 24 | 1 | 4 | 21 | 16 | 2 | 4 | | | | | | | 73 |
| 1.1.1.9 | | 17 | 1 | 1 | | 1 | | | | | | | | | 20 |
| **1.1.2** | | | | | | | | | | | | | | | |
| 1.1.2.1 | | 13 | 1 | 2 | 3 | 3 | 1 | | | | | | | | 23 |
| 1.1.2.2 | | 7 | 1 | | | 1 | | | | | | | | | 9 |
| **1.1.3** | | | | | | | | | | | | | | | |
| 1.1.3.1 | | | | | | | 3 | 2 | 1 | 4 | 3 | 3 | 4 | 1 | 21 |
| 1.1.3.2 | | | | | | 8 | 3 | 1 | 1 | 3 | 1 | | | | 17 |
| 1.1.3.3 | | | | | | | | 1 | 8 | 2 | 2 | 1 | 1 | | 15 |
| **1.2.1** | | | | | | | | | | | | | | | |
| 1.2.1.1 (EAI2_MNL) | | | | | | | 1 | 6 | 3 | 1 | 1 | | | | 12 |
| 1.2.1.2 (EAI2_NTB) | | | | | | | | 2 | 11 | 14 | 22 | 28 | 16 | 1 | 94 |
| 1.2.1.3 | | | | | | | | | | | 1 | | 1 | | 2 |
| **1.2.2** | | 6 | | 5 | 1 | 2 | 2 | 1 | 1 | | | | | | 18 |
| UNCLASSIFIED | | 7 | 2 | | 1 | 1 | | | | | | | | | 11 |
| **TOTAL** | **3** | **151** | **33** | **46** | **44** | **45** | **13** | **18** | **25** | **24** | **30** | **32** | **22** | **2** | **480** |

**Supplementary Table S4** Mean pairwise SNV distances between each sublineage. The numbers in cells with the same row and column label indicate the intragroup mean pairwise distances.

| | 1.1 | 1.1.1 | 1.1.1.1 | 1.1.1.2 | 1.1.13 | 1.1.1.4 | 1.1.1.5 | 1.1.1.6 | 1.1.1.7 | 1.1.1.8 | 1.1.1.9 | 1.1.2 | 1.1.2.1 | 1.1.2.2 | 1.1.3 | 1.1.3.1 | 1.1.3.2 | 1.1.3.3 | 1.2.1 | 1.2.1.1 | 1.2.2.2 | 1.2.1.3 | 1.2.2 |
|---|---|---|---|---|---|---|---|---|---|---|---|---|---|---|---|---|---|---|---|---|---|---|---|
| 1.1 | 570.0 | | | | | | | | | | | | | | | | | | | | | | |
| 1.1.1 | | 451.1 | | | | | | | | | | | | | | | | | | | | | |
| 1.1.1.1 | | | 252.9 | | | | | | | | | | | | | | | | | | | | |
| 1.1.1.2 | | | 530.0 | 340.5 | | | | | | | | | | | | | | | | | | | |
| 1.1.1.3 | | | 473.0 | 549.5 | 431.8 | | | | | | | | | | | | | | | | | | |
| 1.1.1.4 | | | 450.5 | 547.7 | 491.0 | 224.2 | | | | | | | | | | | | | | | | | |
| 1.1.1.5 | | | 478.9 | 575.6 | 517.8 | 464.8 | 323.9 | | | | | | | | | | | | | | | | |
| 1.1.1.6 | | | 464.5 | 560.3 | 502.3 | 456.9 | 478.5 | 135.3 | | | | | | | | | | | | | | | |
| 1.1.1.7 | | | 444.5 | 542.4 | 485.0 | 463.7 | 490.6 | 475.5 | 269.8 | | | | | | | | | | | | | | |
| 1.1.1.8 | | | 446.4 | 544.0 | 487.2 | 465.5 | 493.5 | 477.0 | 372.2 | 174.4 | | | | | | | | | | | | | |
| 1.1.1.9 | | | 459.5 | 557.6 | 500.1 | 479.2 | 505.1 | 492.3 | 457.1 | 459.2 | 284.6 | | | | | | | | | | | | |
| 1.1.2 | | 749.9 | | | | | | | | | | 487.5 | | | | | | | | | | | |
| 1.1.2.1 | | | | | | | | | | | | | 322.0 | | | | | | | | | | |
| 1.1.2.2 | | | | | | | | | | | | | 720.5 | 310.0 | | | | | | | | | |
| 1.1.3 | | 749.6 | | | | | | | | | | 755.0 | | | 502.1 | | | | | | | | |
| 1.1.3.1 | | | | | | | | | | | | | | | | 228.6 | | | | | | | |
| 1.1.3.2 | | | | | | | | | | | | | | | | 671.3 | 131.4 | | | | | | |
| 1.1.3.3 | | | | | | | | | | | | | | | | 635.7 | 672.9 | 138.4 | | | | | |
| 1.2.1 | 800.8 | | | | | | | | | | | | | | | | | | 145.3 | | | | |
| 1.2.1.1 | | | | | | | | | | | | | | | | | | | | 120.33 | | | |
| 1.2.1.2 | | | | | | | | | | | | | | | | | | | | 286.58 | 106.44 | | |
| 1.2.1.3 | | | | | | | | | | | | | | | | | | | | 296.83 | 189.76 | 76.00 | |
| 1.2.2 | 853.6 | | | | | | | | | | | | | | | | | | 846.1 | | | | 430.2 |

**Supplementary Table S5.** Comparisons between experimental spoligotypes and the presence or absence of all 68 DVRs derived from WGS of isolates in each sublineage are listed in a separate sheet in the excel file: **Palittapongarnpim TS5 DR regions vs spoligotypes.xlsx**. The DVRs used for standard experimental spoligotyping are coloured.

**Supplementary Table S6.** List of 35 studies from which spoligotyping data were retrieved for analysis.

| Countries | Regions | Period of sample collection | First Author. Year of Publication | Number of total isolates | Number of L1 isolates | Patient information | Sampling methods |
|---|---|---|---|---|---|---|---|
| Cambodia | Phnom Penh | 2007-2008 | Zhang, C. 2011[5] | 113 | 67 | Both in- and out-patients, various clinical forms | Random samples enriched with MDR isolates |
| Indonesia | Makassar City, South Sulawesi | 2008 | Sasmono, RT. 2012[6] | 179 | 54 | Pulmonary TB | Not explicitly specified |
| Malaysia | Kelantan and Kuala Lumpur | 2009-2013 | Ismail, F. 2014[7] | 220 | 124 | Tertiary hospital TB | Not explicitly specified |
| Myanmar | Yangon | 2002 | Phyu, S. 2009[8] | 310 | 150 | Pulmonary TB, age>14 | Not explicitly specified |
| Philippines | Manila | 1995-1996 | Douglas, JT. 2003[9] | 48 | 39 | Not specified | Random sample |
| | Manila | 2001-2003 | Sia, IG. 2013[10] | 28 | 24 | Pulmonary TB | Index cases and their house hold contacts |
| Singapore | Singapore | 1994 | Sun, YJ. 2004[11] | 68 | 68 | Not specified | Not specified |
| Thailand | Bangkok | 1995-2005 | Yorsangsukkamol, J. 2009[12] | 147 | 42 | TB meningitis | Retrospective hospital based. |
| Vietnam | Ha Not, Ha Tay and Hung Yen | 2003-2005 | Nguyen, VAT. 2012[13] | 221 | 85 | New pulmonary TB | Hospital and population-based |
| | Ho Chi Minh City | 2005-2008 | Duong, DA. 2009[14] | 109 | 41 | Referred fluoroquinolone resistant and control | Consecutive isolates. |
| Taiwan | 4 regions | 2006-2008 | Huang, SF. 2012[15] | 493 | 55 | General, veteran and aboriginal new pulmonary TB | All available culture |
| | Kaohsiung | 2006-2008 | Chang, JR. 2012[16] | 224 | 72 | Pulmonary TB at a referral centre | Not explicitly specified |
| | All regions | 2007-2008 | Chang, CW. 2011[17] | 492 | 29 | MDR-TB | Population-based |
| Bangladesh | Dhaka and Matlab | 2001-2003 | Rahim, Z. 2007[18] | 227 | 99 | Non-hospitalized TB | Not explicitly specified |
| | Matlab, Chittagong | 2001-2007 | Banu, S. 2012[19] | 168 | 109 | Pulmonary TB | Matlab Health and Demographic Surveillance Site |
| | Dhaka | 2002-2005 | Banu, S. 2012[20] | 189 | 59 | Suspected MDR | Referred patients |
| | Sunamganj | 2003-2004 | Storla, DG. 2009[21] | 111 | 97 | Pulmonary TB | All positive culture, 15% of patients |
| India | Multiple sites all over India | 1997-2002 | Gutierrez, MC. 2006[22] | 91 | 41 | Pulmonary TB | Not explicitly specified |
| | Tiruvallur, Tamil Nadu, South India | 1999-2002 | Narayanan, S. 2008[23] | 1215 | 1026 | Pulmonary TB | Not explicitly specified |
| | Tiruvallur, Tamil Nadu, South India | 1999-2003 | Shanmugam, S. 2011[24] | 1649 | 978 | Pulmonary TB | Not explicitly specified |
| | Kerala, South India | 1998-2005 | Joseph, BV. 2013[25] | 168 | 113 | Repository of isolates subjected to drug susceptibility testing | Not explicitly specified |

| | | | | | | | |
|---|---|---|---|---|---|---|---|
| | Hyderabad and rural Andhra Pradesh, South India | 2000-2005 | Thomas, SK 2011[26] | 101 | 38 | Serviced samples and MDR collections | Not explicitly specified |
| | 8 centres In India | 2001-2003 | Singh, UB. 2007[27] | 540 | 187 | Mostly pulmonary TB | Random selection |
| | Delhi, North India | 2005-2006 | Varma-Basil, M. 2011[28] | 101 | 27 | Pulmonary TB in a referral centre | Not explicitly specified |
| | Ghatampur, Uttar Pradesh, North India | 2005-2007 | Sharma, P. 2017[29] | 335 | 64 | Collection as a part of an immunotherapeutic trial | All cases in specified areas. |
| | 9 regions in India | 2007-2010 | Singh, J. 2015[30] | 628 | 152 | Mostly pulmonary TB | Random sampling |
| | Bhopal, Madhya Pradesh, Central India | 2007-2011 | Desikan, P. 2016[31] | 340 | 86 | Mostly pulmonary TB | Convenient samples |
| | Varanasi, Uttar Pradesh, North India | 2008-2010 | Gupta, A. 2014[32] | 104 | 22 | Pulmonary TB | Not explicitly specified |
| | Assam, East India | 2010-2013 | Devi, KR. 2015[33] | 189 | 29 | Pulmonary TB | Not explicitly specified |
| Nepal | Kathmandu | 2009-2010 | Malla, B. 2012[34] | 261 | 30 | New and treated cases | Convenient sample |
| Pakistan | Karachi and 4 provinces | 2003-2005 | Tanveer, M. 2008[35] | 926 | 42 | Mostly pulmonary TB | Serviced samples |
| Mozambique | 7 provinces | 2007-2008 | Viegas, S. 2010[36] | 445 | 132 | Samples from drug resistance surveillance study | Consecutive enrolment |
| Tanzania | Mwanza city, north-western Tanzania | Before 2014 | Stavrum, R. 2014[37] | 252 | 42 | Pulmonary TB, residence | Cohort |
| | Serengeti ecosystem | 2010-2012 | Mbuki, EV. 2015[38] | 214 | 25 | New TB cases | Consecutive enrollment |
| | Serengeti ecosystem and Dar es Salaam | Before 2016 | Mbuki, EV. 2016[39] | 293 | 49 | Field collection | Not explicitly specified |

**Supplementary Table S7.** The numbers of isolates with different EAI spoligotypes in various countries in Asia and East Africa. The numbers of isolates are shown up to SIT2000. The numbers of isolates with SIT numbers beyond that were small. The number of isolates in homoplastic spoligotype such as SIT48, SIT236 etc. were italic and excluded from analysis. The bold and underlined numbers indicate the numbers of isolates in the most common SIT. The numbers of the second and third common SIT are shown in bold and underlined respectively. The total number of listed isolates and all EAI isolates are indicated in the last two rows.

| SIT | Mozambique[36] | Tanzania, northwestern[37] | Tanzania, northern (2015)[38] | Tanzania, northern (2016)[39] | Nepal[34] | Pakistan[35] | India, multiple sites (2006)[22] | India multiple sites (2007)[27] | India multiple sites (2015)[30] | Northern India, Ghatampur[29] | Northern India, Varanasi[32] | Northern India, Delhi[28] | Central India, Bhopal[31] | Southern India, MDR[26] | Southern India, Kerala [25] | Southern India, Tiruvallur (2008)[23] | Southern India, Tiruvallur (2011)[24] | East India, Assam[33] | Bangladesh, Matlab[19] | Bangladesh, Dhaka and Matlab[18] | Bangladesh, Dhaka, MDR[20] | Bangladesh, Sunamgnaj[21] | Myanmar[8] | This study | Thailand, meningitis[12] | Cambodia[5] | Vietnam, multiple sites[13] | Southern Vietnam, Ho Chi Minh | Malaysia, peninsular [7] | Singapore[11] | Indonesia, Makassar[6] | Philippines (2003)[9] | Philippines (2013)[10] | Southern Taiwan[16] | Taiwan, MDR[17] | Taiwan, multiple sites[15] |
|---|---|---|---|---|---|---|---|---|---|---|---|---|---|---|---|---|---|---|---|---|---|---|---|---|---|---|---|---|---|---|---|---|---|---|---|---|
| **6** | | | | | | | | | | | | | | 1 | 4 | | 3 | | | | | | 2 | | | | | 1 | | | | | | | | |
| **8** | 12 | **7** | **4** | **12** | | | | | 3 | 1 | | | | | | 18 | 12 | | | | | | | 1 | | | | | | | | | | | | |
| **10** | | | | 1 | | | | | | | | | | | | | | | | | | | | | | | | | | | | | | | | |
| **11** | 1 | | | | **4** | **15** | **27** | **88** | **32** | **31** | **4** | **11** | **62** | **6** | **48** | **336** | **445** | 3 | **5** | 7 | 1 | | 7 | 2 | | | | | 1 | **9** | | | | | 2 | |
| **16** | | | | | | | | | | | | | | | | | 11 | | | | | | | | | | | | | | | | | | | |
| **19** | | | | | 1 | | | | | | | | | | | | 2 | | | | | | | 9 | **5** | 2 | | 1 | 9 | **17** | **13** | **33** | **18** | **62** | **19** | **45** |
| **43** | | | | | | | | | | | | | | | 1 | 40 | 38 | | | | | | | | | | | | | | | | | | | |
| ***48*** | *30* | | | *1* | *1* | | *1* | *10* | *3* | *3* | | *3* | *2* | | *1* | *37* | *45* | | *3* | *9* | *3* | *1* | *4* | *20* | | *9* | | | *1* | | *5* | | | | | |
| **72** | | | | | | | | | | | | | | | | | 2 | | | | 1 | | 4 | | | | | | | | | | | | | |
| **89** | | | | | | | | | | | | | | | | | 2 | | | | | | **11** | **83** | **8** | **6** | | | 5 | 1 | | | | 1 | | |
| **96** | | | | | | | | | | | | | | | | | | | 2 | 2 | | 2 | | | | | | | | | | | | | | |
| **109** | | | | | | | | | | | | | | | | | 2 | | | | | | | | | | | | | | | | | | | |
| **113** | | | | | | | | | | | | | | | | 4 | 3 | | | | | | 1 | | | | | | | | | | | | | |
| **126** | | **29** | **20** | **18** | | **4** | | **20** | 7 | 1 | | 1 | | **7** | 3 | **80** | 91 | | | | | | 4 | | | | | | 1 | | | | | | | |
| **129** | **14** | | | | | | | | | | | | | | | | | | | | | | | | | | | | | | | | | | | |
| **138** | | | | | **10** | | 2 | 7 | 6 | **6** | 2 | | | | 1 | 6 | 2 | 3 | 3 | **8** | **5** | | 1 | 3 | | | | | | | | | | | | |
| **139** | | | | | | | | | | | | | | | | | | | | | | | 1 | 2 | 3 | **4** | **41** | **25** | | 1 | | | | | | |
| **152** | | | | | | | | | | | | | | | | | | | | | | | | | | | **6** | | | | | | | | | |
| **177** | | | | | | | | | | | | | | | | | 2 | | | | | | | | | | | | | | | | | | | |
| ***204*** | | | | | | | | | | *1* | | | | | | | | | | | | | | *3* | *1* | *7* | | | | | | | | | | |
| **234** | | | | | | | | | | | | | | | | | | | | | | | 1 | | | | 1 | 1 | | | | | | | | |
| **235** | | | | | | | | | | | | | | | | 2 | | | | | | | | | | | | | | | | | | | | |
| ***236*** | | | | | | *15* | | *10* | *46* | *2* | | *3* | | *1* | *3* | *24* | *26* | *1* | *1* | *5* | | *1* | *6* | *60* | *3* | *6* | *7* | *7* | *10* | | *2* | | | | | |
| **256** | | | | | | | | | 2 | 1 | | | | | | | | | | | | | 2 | **58** | **5** | 2 | | | 12 | 1 | **6** | | | | | |
| **270** | | | | | | | | 2 | | | | | | | 1 | | | | | | | | | | | | | | | | | | | | | |
| **286** | | | | | | | | | | | | | | | | | | | | | | | | | | | | | | | | | 1 | | | |
| **287** | | | | | | | | | | | | | | | | | 1 | | | | | | 1 | 1 | | | | | | | 3 | | | | | 1 |
| **288** | | | | 2 | | | 4 | | | | | | | | | | | | | | | | | | | | | | | | | | | | | |
| **292** | | | | | 1 | | | | 1 | | | 1 | | | | 2 | 2 | 1 | **13** | **11** | 2 | **23** | **28** | 13 | | | | | | | | | | | | |
| **298** | | | | | | | | | | | | | | | | | 2 | | 1 | | | | | | | | | | 1 | | | | | | | |
| **299** | | | | | | | | | | | | | | | **5** | | | | | | | | | | | | | | | 1 | | | | | | |
| **337** | | | | | | | | | | | | | | | | 3 | | | | | | | | | | | | | | | | | | | | |
| **338** | | | | | | | | | | | | | | | | | 2 | | | | | | | | | | | | | | | | | | | |
| **340** | | | | | | | 1 | 3 | 3 | | | | | | 4 | 75 | **109** | 1 | | | | | 4 | | | | | | 1 | | | | | | | |
| **342** | | | | | | | 2 | | | | | | | | | | 2 | | 0 | | | | 1 | 1 | | | | | | | | | | | | |
| **346** | | | | | | | | | | | 1 | | | | | | | | | | | | | | | | | | | | | | | | | |
| **349** | 2 | | | | | | | | | | | | | | 1 | | | | 1 | 2 | | | 2 | 3 | | | | | 1 | | | | | | | |
| **355** | | | | | | | | 10 | 2 | | | | | | **5** | 19 | 27 | | | | | | 1 | 1 | | | | | | 1 | | | | | | |
| **380** | | | | | | | | | | | 1 | | | | | 3 | 2 | 1 | | | | | | | | | | | | | | | | | | |
| **413** | | | | | | | | | | | | | | | | | 2 | | | | | | | 1 | | | | | | | | | | | | |
| **414** | | | | | | | | | | | | | | | | 2 | 2 | | | | | | | | | | | | | 1 | | | | | | |
| **458** | | | | 1 | | | | 2 | | | | | | | | | | | | | | | | | | | | | | | | | | | | |
| **459** | | | | | | | | | | | | | | | | | | | | | | | | | | 6 | | | | | | | | | | |
| **460** | | | | | | | | | | | | | | | | | 2 | | | | | | | | | | | | | | | | | | | |
| **463** | | | | | | | | | | | | | | | | | | | | | | | 1 | | | | | | | | | | | | | |
| **473** | | | | | | | | | 2 | | | | | | | 6 | 11 | | | | | | 1 | | | | | | | 1 | | | | | | |
| **474** | | | | | | | | | | | 1 | | | | | 8 | 10 | | | | | | | | 1 | | | | | | | | | | | |
| **477** | | | | | | | | | | | | | | | | | | 1 | | | | | | 2 | | | | | | | | | | | | |
| **483** | | | | | | | | | | | | | | | | | 1 | | | | | | 1 | 1 | | | | | 1 | | | 1 | | 1 | 1 | |
| **493** | | | | | | | | | | | | | | | | | | | | | | | 1 | 15 | | | | | | | | | | | | |
| **514** | | | | | | | | | | | | | | | | | | | | | | | | 2 | | | | | | 1 | | | | | | |
| ***517*** | | | | | | | | | | | | | | | | | | *1* | | | | | | *3* | | | | | | | | | | | | |
| **523** | | | | 1 | | | | | | | | | | | | | | | | | | | | 2 | | | | | | | | | | | | |
| **529** | | | | | | | | | | | | | | | | | | | | | | | | 1 | | | | | | | | | | | | |
| **564** | | | | | | | | | | | | | | | | | | | | | | | | | | | 2 | | | | | | | | | |
| **591** | | | | | | | | 3 | 3 | | 1 | 1 | | 4 | 2 | 31 | 44 | 1 | 2 | 3 | | | 5 | 22 | | | | | **13** | 1 | | | | | | |
| **617** | | | | | | | | | | | | | | | | | | | | | | | | | | | 3 | | | | | | | | | |
| ***618*** | | | | | | | | | | | | | | | | | | | | | | | | *3* | | | *1* | | | | *1* | | | | | |
| **624** | | | | | | | | | | | | | | | | 3 | 2 | | | | | | | | | | | | | | | | | | | |
| **625** | | | | | | | | 2 | | | | | | | 1 | | 2 | | | | | | | | | | | | | | | | | | | |
| **629** | | | | | | | | | | | | | | | | 2 | | | | | | | | | | | | | | | | | | | | |
| **652** | | | | | | | | | | | | | | | | | 2 | | | | | | | | | | | | | | | | | | | |
| **654** | | | | | | | | | 2 | | | | | | | | | | | | | | 1 | | | | | | | | | | | | | |
| **702** | 11 | | | 1 | | | | | | | | | | | | | | | | | | | | | | | | | | | | | | | | |
| **711** | | | | | | | | | | | | | | | 3 | | | | | | | 1 | | | | | | | | | | | | | | |

| | | | | | | | | | | | | | | | | | | | | | | | | | | | | | | | | | | | | |
|---|---|---|---|---|---|---|---|---|---|---|---|---|---|---|---|---|---|---|---|---|---|---|---|---|---|---|---|---|---|---|---|---|---|---|---|---|
| **730** | | | | | | | | | | | | | | | | | 3 | | | | | | | | | | | | | | | | | | | |
| **733** | | | | | | | | | | | | | | | | | 17 | | | | | | | | | | | | 1 | | | | | | | |
| **735** | | | | | | | | | | | | | | | | 2 | | | | | | | 0 | 1 | 1 | | | | | | | | | | | |
| **745** | | | | | | | | | | | | | | | | | | | | | | | 0 | 1 | | 0 | | | <u>**33**</u> | | | | | | | |
| **758** | | | | | | | | | | | | | | | | | | | | | | | | | | | | | | | | 1 | | | | 1 |
| **763** | | | | | | | | | | | | | | | | 2 | 2 | | | | | | 1 | | | | | | | | | | | | | |
| **773** | | | | | | | | | | | | | | | | | | | | | | | | 1 | | | | | | | | | | | | |
| **786** | | | | | | | | | | | | | | | | | | | | | | | | 1 | | | | | | | | | | | | |
| **792** | | | | | | | | | | | | | | | | | | | | | | | | 2 | | | | | | | 1 | | | | | |
| **806** | **13** | | | | | | | | | | | | | | | 3 | 3 | 1 | | | | | | | | | | | | | | | | | | |
| **821** | | | | | | | | | | | | | | | | | | | | | | | | | | | | | | | | | 1 | | | |
| **882** | | | | | | | | | | | | | | | | | 2 | | <u>4</u> | 6 | 1 | 3 | | | | | | | | | | | | | | |
| **889** | | | | | | | | | | | | | | | | | 1 | | | | | | | | | | | | | | | | | | | |
| **892** | | | | | | | | | | | | | | | | | | | | | | | | | | | | | | | 1 | | | | | |
| **894** | | | | | | | | | | | | | | | | | | | | | | | | | | | | | | 1 | 3 | | | 1 | 5 | 3 |
| **895** | | | | | | | | | | | | | | | | | | | | | | | | | | | | | | | | 1 | | | | |
| **912** | | | | | | | | | | | | | | | | | | | | | | | | 1 | | | | | | | | | | | | |
| **924** | | 1 | | | | | | | | | | | | | | | | | | | | | | | | | | | | | | | | | | |
| **934** | | | | | | | | 2 | | | | | | | | | 2 | | | | | | | 1 | | | | | | | | | | | | |
| **937** | | | | | | | | | | | | | | | | | | | | | | | | 4 | | | | | | | | | | | | |
| **939** | | | | | | | | | 1 | | | | | | | | | | | | | | | 15 | | | | | 1 | | | | | | | |
| **944** | | | | | | | | | | | | | | | | | | | | | | | | 3 | | | | | | | | | | | | |
| ***947*** | | | | | | | | | *3* | | | | | | | | *2* | | | | | | *3* | *8* | | | | | | | *1* | | | | | |
| **951** | | | | | | | | | | | | | | | | | | | | | | | 4 | 14 | 3 | 1 | | | 4 | 1 | | | | | | |
| **962** | | | | | | | | | | | 1 | | | | | | 2 | | | | | | | | 2 | | | | 1 | | | | | | | |
| **974** | | | | | | | | | | | | | | | | | | | | | | | | | | | | | | | | | | | | |
| **1062** | 4 | | | | | | | | | | | | | | | | | | | | | | | | | | | | | | | | | | | |
| **1090** | | | | 1 | | | | | | | | | | | 1 | | 2 | | | | | | | | | | | | | | | | | | | |
| **1097** | | | | | | | | | | | | | | | | | | | | | | | 1 | | | | | | | | | | | | | |
| **1169** | | | | | | | | | | | | | | | | | | | | | | | | | | | | | | | | | | | | 1 |
| **1171** | | | | | | | | | | | | | | | | | | | | | | | | | | | | | | | | | | | 1 | |
| **1182** | | | | | | | | | | 1 | | | | | | | | | | | | | | 1 | | | | | | | | | | | | |
| **1183** | | | | | | | | | | | | | | | | | | | | | | | | 0 | | | | | 1 | | | | | | | |
| **1251** | 2 | | | | | | | | | | | | | | | | | | 1 | | 4 | | | 1 | | | | | | | | | | | | |
| **1263** | | | | | | | | | | | | | | | 1 | | | | | | | | | | | | | | | | | | | | | |
| **1274** | | | | | | | | | | | | | | | | | | | | | | | | 12 | | | | | | | | | | | | |
| **1313** | | | | | | | | | | | | | | | | | | | | | | | | 1 | | | | | | | | | | | | |
| **1316** | | | | | | | | | | | | 1 | | | | | 3 | 1 | | | | | | 1 | | | | | | | | | | | | |
| **1326** | | | | | | | | | | 1 | | | | | | | | | | | | | | | | | | | | | | | | | | |
| **1342** | | | | | | | | | 1 | | | 2 | | | | | | | | | | | | | | | | | | | | | | | | |
| **1365** | | | | | | | | | | | | | | | | | 2 | | | | | | | | | | | | | | | | | | | |
| **1369** | | 2 | | 1 | | | | | 3 | 1 | | | | | | | 2 | | | | | | | | | | | | | | | | | | | |
| **1372** | | | | | | | | | | | | | | | | | | | | | | | | 1 | | | 1 | | 1 | | | | | | | |
| **1373** | | | | | | | | | | | 1 | | | | | | | | | | | | | | | | | | | | | | | | | |
| **1375** | | | | | | | | | | | | | | | | | | | | | | | | | | | | | | | | | | | | |
| **1378** | | | | | | | | | | | | | | | | | | | | | | | | | | | | | | | | | | | | |
| **1388** | | | | | | | | | | | | | | | | | | | | | | 2 | | | | | | | | | | | | | | |
| **1389** | | | | | | | | | | | | | | | | | 2 | 1 | | 2 | | 1 | | | | | | | | | | | | | | |
| **1390** | | | | | | | | | | | 1 | | | | | | 2 | | | | | | 1 | 1 | | | | | | | | | | | | |
| **1391** | | | | | | | 1 | | | | | | | | 1 | | | | 2 | | | 1 | 3 | 1 | | | | | | | | | | | | |
| **1395** | | | | | | | | | | | | | | | | | | | | | | | | | | | | | 1 | | | | | | | |
| **1396** | | | | | | | | | | | | | | | | | 1 | | | | | | | | | | | | | | | | | | | |
| **1400** | | | | | | | | | | | | | | | | | | | 1 | 3 | | | | | | | | | | | | | | | | |
| **1404** | | | | | | | | | | | | | | | | | | | | | | | | 1 | | | | | | | | | | | | |
| **1406** | | | | | | | | | | | | | | | | | | | | 2 | | | | | | | | | | | | | | | | |
| **1409** | | | | | | | | | | | | | | | | | | | 2 | | | | 2 | | | | | | | | | | | | | |
| **1414** | | | | | | | | | | | | | | | | | | | | | | | | | | | | | | | | | | | | |
| **1417** | | | | | | | | | | | | | | | | | 1 | | | | | | | | | | | | | | | | | | | |
| **1425** | | | | | | | | | | | | | | | | | | | | | | 1 | 1 | | | | | | | | | | | | | |
| **1427** | | | | | | | | | | | | | | | | | | | 1 | | | | | | | | | | | | | | | | | |
| **1479** | | | | | | | | | | | | | | | | | | | | | | | | | | | | | | 1 | | | | | | |
| **1490** | | | | | | | | | | | | | | | | | | | | | | | 3 | 1 | | | | | 1 | | | | | | | |
| **1493** | | | | | | | | | | | | | | | | | | | | | | | | | | | | | | 1 | | | | | | |
| **1495** | | | | | | | | | | | | | | | | | | 1 | | | | | | | | | | | | | | | | | | |
| **1508** | | | | | | | | | | | | | | | | | | | | | | | | | | | | | | | 1 | | | | | |
| **1509** | | | | | | | | | | | | | | | | | | | | | | | | | | | | | | | | 1 | | | | |
| **1510** | | | | | | | | | | | | | | | | | | | | | | | | | | | | | | | | 1 | | | | |
| **1511** | | | | | | | | | | | | | | | | | | | | | | | | | | | | | | 1 | | | | | | |
| **1512** | | | | | | | | | | | | | | | | | 1 | | | | | | 1 | | | | | | 1 | | | | | | | |
| **1680** | | | | | | | | | | | | | | | | | 2 | | | | | | | | | | | | | | | | | | | |
| **1731** | | | | | | | | | | | | | | | | | | | | | | | | | | | 1 | | | | | | | | | |
| **1734** | | | | | 1 | | | | | | | | | | | | | | | | 1 | | | | | | | | | | | | | | | |
| **1781** | | | | | | | | | | | | | | | | | | | | | | | | | | | | | | | 1 | | | | | 1 |
| **1801** | | | 1 | 1 | | | | | 1 | | | | | | | | | | | | | | | 1 | | | | | | | | | | | | |
| **1866** | | | | | | | | | | | | | | | | | 1 | | | | | 1 | | | | | | | | | | | | | | |
| **1875** | | | | | | | | | | | | | | | | | | | | | | | 3 | 1 | | | | | | | | | | | | |
| **1886** | | | | | | | | | | | | | | | | | | | | | | | | | | | | | | 1 | | | | | | |
| **1901** | | | | | | | | | | | | | | | | | | | | | | | | | | | 2 | | | | | | | | | |
| **1903** | | | | | | | | | | | | | | | | | | | | | | | | | | | 3 | | | | | | | | | |
| **1944** | | | | | | | | 2 | | | | | | | | | | | | | | | | | | | | | | | | | | | | |
| **1948** | | | | | | | | 1 | | | | | | | | | | | | | | | | | | | | | | | | | | | | |
| **1956** | | | | | | | | | | | | | | | 1 | | | | | | | | | | | | | | | 2 | | | | | | |
| **1957** | | | | 1 | | | | 2 | | | | | | | | | | | | | | | | | | | | | | | | | | | | |
| **1966** | | | | | | | | 2 | | | | | | | | | | | | | | | | | | | | | | | | | | | | |
| **1970** | | | | | | | | | 2 | 1 | | 1 | | 1 | | | | | | | | | | | | | | | | | | | | | | |
| **1977** | | | | | | | | 2 | | | | | | | | | | | | | | | | | | | | | | | | | | | | |
| Number of Listed Isolates | 89 | 39 | 25 | 40 | 18 | 34 | 38 | 166 | 123 | 49 | 13 | 24 | 64 | 20 | 87 | 708 | 964 | 17 | 42 | 60 | 18 | 37 | 114 | 387 | 32 | 43 | 68 | 35 | 101 | 43 | 38 | 38 | 20 | 65 | 28 | 52 |
| **Total EAI** | **132** | **42** | **25** | **49** | **30** | **42** | **41** | **187** | **152** | **64** | **22** | **27** | **86** | **38** | **113** | **1026** | **978** | **29** | **109** | **99** | **59** | **97** | **150** | **480** | **42** | **67** | **85** | **41** | **124** | **65** | **54** | **39** | **24** | **72** | **29** | **55** |

**Supplementary Table S8.** Correlation coefficients of the frequencies of spoligotypes between studies in various countries. The yellow colour highlights the high correlation coefficients (>0.8) of studies in the same country. The orange colour highlights the high correlation coefficients between studies in neighbouring countries. The intermediate correlation coefficients are highlighted with light orange (0.6-0.8) or blue (0.4-0.6). The low correlation coefficients (0.2-0.4) are highlighted with grey. There were high correlations between ISEA countries, Singapore and Taiwan as well as between Pakistan and India, and, between Bangladesh and Myanmar.

| Countries | Mozambique[36] | Tanzania, northwestern[37] ) | Tanzania, northern (2015)[38] | Tanzania, northern (2016)[39] | Nepal[34] | Pakistan[35] | India, multiple sites (2006)[22] | India multiple sites (2007)[27] | India multiple sites (2015)[30] | Northern India, Ghatampur[29] | Northern India, Varanasi[32] | Northern India, Delhi[28] | Central India, Bhopal[31] | Southern India, MDR[26] | Southern India, Kerala[25] | Southern India, Tiruvallur (2008)[23] | Southern India, Tiruvallur (2011)[24] | East India, Assam[33] | Bangladesh, Matlab[19] | Bangladesh, Dhaka and Matlab[18] | Bangladesh, Dhaka, MDR[20] | Bangladesh, Sunamgnaj[21] | Myanmar[8] | This study | Thailand, meningitis[12] | Cambodia[5] | Vietnam, multiple sites[13] | Southern Vietnam, Ho Chi Minh City[14] | Malaysia, peninsular [7] | Singapore[11] | Indonesia, Makassar[6] | Philippines (2003)[9] | Philippines (2013)[10] | Southern Taiwan[16] | Taiwan, MDR[17] | Taiwan, multiple sites[15] |
|---|---|---|---|---|---|---|---|---|---|---|---|---|---|---|---|---|---|---|---|---|---|---|---|---|---|---|---|---|---|---|---|---|---|---|---|---|
| Mozambique[36] | 1 | | | | | | | | | | | | | | | | | | | | | | | | | | | | | | | | | | | |
| Tanzania[37] | 0.091 | 1 | | | | | | | | | | | | | | | | | | | | | | | | | | | | | | | | | | |
| Tanzania [38] | 0.074 | 0.995 | 1 | | | | | | | | | | | | | | | | | | | | | | | | | | | | | | | | | |
| Tanzania [39] | 0.255 | 0.927 | 0.914 | 1 | | | | | | | | | | | | | | | | | | | | | | | | | | | | | | | | |
| Nepal[34] | -0.011 | -0.015 | -0.014 | -0.020 | 1 | | | | | | | | | | | | | | | | | | | | | | | | | | | | | | | |
| Pakistan[35] | 0.018 | 0.241 | 0.244 | 0.199 | 0.346 | 1 | | | | | | | | | | | | | | | | | | | | | | | | | | | | | | |
| India, multiple[22] | 0.017 | -0.013 | -0.012 | -0.004 | 0.419 | 0.949 | 1 | | | | | | | | | | | | | | | | | | | | | | | | | | | | | |
| India multiple[27] | 0.012 | 0.200 | 0.203 | 0.165 | 0.413 | 0.988 | 0.953 | 1 | | | | | | | | | | | | | | | | | | | | | | | | | | | | |
| India multiple[30] | 0.046 | 0.210 | 0.205 | 0.200 | 0.496 | 0.959 | 0.936 | 0.972 | 1 | | | | | | | | | | | | | | | | | | | | | | | | | | | |
| Northern India[29] | 0.031 | 0.028 | 0.025 | 0.027 | 0.525 | 0.953 | 0.975 | 0.964 | 0.966 | 1 | | | | | | | | | | | | | | | | | | | | | | | | | | |
| Northern India[32] | -0.011 | -0.024 | -0.022 | -0.033 | 0.627 | 0.743 | 0.783 | 0.773 | 0.798 | 0.825 | 1 | | | | | | | | | | | | | | | | | | | | | | | | | |
| Northern India[28] | 0.012 | 0.072 | 0.073 | 0.053 | 0.351 | 0.955 | 0.947 | 0.951 | 0.941 | 0.949 | 0.755 | 1 | | | | | | | | | | | | | | | | | | | | | | | | |
| Central India[31] | 0.023 | -0.009 | -0.009 | -0.013 | 0.360 | 0.966 | 0.983 | 0.964 | 0.939 | 0.979 | 0.772 | 0.966 | 1 | | | | | | | | | | | | | | | | | | | | | | | |
| Southern India, MDR[26] | -0.008 | 0.664 | 0.671 | 0.556 | 0.200 | 0.746 | 0.574 | 0.728 | 0.728 | 0.596 | 0.516 | 0.667 | 0.587 | 1 | | | | | | | | | | | | | | | | | | | | | | |
| Southern India[25] | 0.013 | 0.044 | 0.046 | 0.030 | 0.365 | 0.959 | 0.964 | 0.971 | 0.946 | 0.961 | 0.762 | 0.950 | 0.978 | 0.635 | 1 | | | | | | | | | | | | | | | | | | | | | |
| Southern India[23] | 0.036 | 0.215 | 0.217 | 0.192 | 0.347 | 0.964 | 0.930 | 0.969 | 0.960 | 0.929 | 0.744 | 0.931 | 0.939 | 0.736 | 0.957 | 1 | | | | | | | | | | | | | | | | | | | | |
| Southern India[24] | 0.022 | 0.179 | 0.181 | 0.152 | 0.336 | 0.961 | 0.934 | 0.966 | 0.956 | 0.929 | 0.744 | 0.934 | 0.944 | 0.720 | 0.961 | 0.997 | 1 | | | | | | | | | | | | | | | | | | | |
| East India[33] | 0.077 | -0.027 | -0.026 | -0.039 | 0.756 | 0.552 | 0.611 | 0.604 | 0.669 | 0.670 | 0.727 | 0.598 | 0.576 | 0.396 | 0.588 | 0.600 | 0.600 | 1 | | | | | | | | | | | | | | | | | | |
| Bangladesh[19] | -0.018 | -0.023 | -0.022 | -0.033 | 0.356 | 0.299 | 0.322 | 0.312 | 0.349 | 0.338 | 0.318 | 0.380 | 0.313 | 0.217 | 0.309 | 0.300 | 0.299 | 0.458 | 1 | | | | | | | | | | | | | | | | | |
| Bangladesh[18] | -0.019 | -0.025 | -0.023 | -0.035 | 0.617 | 0.376 | 0.416 | 0.411 | 0.470 | 0.469 | 0.495 | 0.443 | 0.394 | 0.281 | 0.393 | 0.383 | 0.380 | 0.655 | 0.887 | 1 | | | | | | | | | | | | | | | | |
| Bangladesh, MDR[20] | 0.016 | -0.020 | -0.019 | -0.028 | 0.741 | 0.122 | 0.176 | 0.172 | 0.244 | 0.260 | 0.361 | 0.143 | 0.130 | 0.058 | 0.132 | 0.124 | 0.115 | 0.530 | 0.479 | 0.599 | 1 | | | | | | | | | | | | | | | |
| Bangladesh[21] | -0.025 | -0.014 | -0.013 | -0.020 | 0.075 | -0.013 | -0.013 | -0.017 | 0.007 | -0.015 | -0.027 | 0.071 | -0.011 | -0.020 | -0.015 | -0.014 | -0.015 | 0.173 | 0.871 | 0.671 | 0.282 | 1 | | | | | | | | | | | | | | |
| Myanmar[8] | -0.038 | 0.092 | 0.095 | 0.063 | 0.155 | 0.217 | 0.198 | 0.218 | 0.252 | 0.196 | 0.164 | 0.279 | 0.197 | 0.244 | 0.214 | 0.243 | 0.243 | 0.311 | 0.815 | 0.643 | 0.277 | 0.833 | 1 | | | | | | | | | | | | | |
| This study | -0.037 | -0.023 | -0.021 | -0.029 | 0.022 | -0.005 | -0.004 | -0.003 | 0.043 | 0.015 | 0.019 | 0.018 | 0.000 | 0.057 | -0.003 | 0.003 | 0.006 | 0.042 | 0.100 | 0.088 | 0.022 | 0.092 | 0.413 | 1 | | | | | | | | | | | | |
| Thailand, meningitis[12] | -0.040 | -0.022 | -0.021 | -0.032 | 0.013 | -0.021 | -0.023 | -0.028 | -0.010 | -0.010 | 0.007 | -0.027 | -0.017 | -0.032 | -0.029 | -0.029 | -0.025 | -0.051 | -0.043 | -0.046 | -0.037 | -0.026 | 0.252 | 0.811 | 1 | | | | | | | | | | | |
| Cambodia[5] | -0.036 | -0.020 | -0.019 | -0.028 | -0.005 | -0.019 | -0.021 | -0.025 | -0.020 | -0.015 | -0.039 | -0.024 | -0.015 | -0.029 | -0.026 | -0.028 | -0.026 | -0.045 | -0.038 | -0.041 | -0.033 | -0.023 | 0.203 | 0.594 | 0.707 | 1 | | | | | | | | | | |
| Vietnam[13] | -0.024 | -0.013 | -0.013 | -0.019 | -0.016 | -0.013 | -0.014 | -0.017 | -0.021 | -0.014 | -0.026 | -0.016 | -0.010 | -0.019 | -0.017 | -0.019 | -0.019 | -0.030 | -0.026 | -0.028 | -0.022 | -0.015 | -0.001 | -0.009 | 0.233 | 0.386 | 1 | | | | | | | | | |
| Southern Vietnam[14] | -0.019 | -0.010 | -0.010 | -0.015 | -0.009 | -0.010 | -0.011 | -0.013 | -0.017 | -0.011 | -0.020 | -0.013 | -0.008 | -0.011 | -0.010 | -0.014 | -0.015 | -0.024 | -0.020 | -0.021 | -0.017 | -0.012 | 0.010 | 0.001 | 0.259 | 0.405 | 0.980 | 1 | | | | | | | | |
| Malaysia[7] | -0.036 | 0.004 | 0.005 | -0.009 | 0.005 | 0.012 | 0.004 | 0.016 | 0.047 | 0.013 | 0.050 | 0.031 | 0.010 | 0.137 | 0.016 | 0.036 | 0.039 | 0.039 | 0.015 | 0.029 | -0.032 | -0.025 | 0.095 | 0.344 | 0.319 | 0.168 | -0.023 | -0.009 | 1 | | | | | | | |
| Singapore[11] | -0.018 | -0.020 | -0.019 | -0.029 | 0.228 | 0.432 | 0.438 | 0.433 | 0.422 | 0.437 | 0.336 | 0.431 | 0.450 | 0.269 | 0.446 | 0.420 | 0.426 | 0.240 | 0.121 | 0.158 | 0.033 | -0.023 | 0.092 | 0.129 | 0.428 | 0.216 | 0.028 | 0.069 | 0.226 | 1 | | | | | | |
| Indonesia[6] | -0.032 | -0.018 | -0.017 | -0.025 | 0.059 | -0.017 | -0.019 | -0.022 | -0.005 | -0.006 | -0.035 | -0.022 | -0.014 | -0.026 | -0.023 | -0.025 | -0.022 | -0.041 | -0.034 | -0.037 | -0.030 | -0.021 | -0.012 | 0.260 | 0.522 | 0.234 | -0.020 | 0.020 | 0.298 | 0.767 | 1 | | | | | |
| Philippines[9] | -0.019 | -0.011 | -0.010 | -0.015 | 0.080 | -0.010 | -0.011 | -0.013 | -0.017 | -0.011 | -0.021 | -0.013 | -0.008 | -0.015 | -0.014 | -0.015 | -0.011 | -0.024 | -0.020 | -0.022 | -0.018 | -0.012 | -0.025 | 0.063 | 0.416 | 0.189 | -0.012 | 0.031 | 0.216 | 0.861 | 0.861 | 1 | | | | |
| Phillipines[10] | -0.018 | -0.010 | -0.010 | -0.014 | 0.080 | -0.010 | -0.011 | -0.013 | -0.017 | -0.011 | -0.020 | -0.012 | -0.008 | -0.015 | -0.013 | -0.014 | -0.011 | -0.023 | -0.020 | -0.021 | -0.017 | -0.012 | -0.025 | 0.063 | 0.416 | 0.190 | -0.011 | 0.031 | 0.216 | 0.860 | 0.860 | 0.995 | 1 | | | |
| Southern Taiwan[16] | -0.017 | -0.010 | -0.009 | -0.014 | 0.081 | -0.009 | -0.010 | -0.012 | -0.016 | -0.010 | -0.019 | -0.012 | -0.007 | -0.014 | -0.013 | -0.014 | -0.010 | -0.022 | -0.019 | -0.020 | -0.016 | -0.011 | -0.017 | 0.078 | 0.429 | 0.201 | -0.011 | 0.032 | 0.220 | 0.865 | 0.866 | 0.998 | 0.997 | 1 | | |
| Taiwan, MDR[17] | -0.020 | -0.013 | -0.012 | -0.019 | 0.111 | 0.086 | 0.087 | 0.083 | 0.076 | 0.086 | 0.054 | 0.083 | 0.092 | 0.042 | 0.083 | 0.078 | 0.082 | 0.031 | 0.008 | 0.015 | -0.007 | -0.015 | -0.008 | 0.057 | 0.395 | 0.178 | -0.015 | 0.027 | 0.207 | 0.885 | 0.877 | 0.959 | 0.957 | 0.964 | 1 | |
| Taiwan[15] | -0.019 | -0.011 | -0.010 | -0.015 | 0.080 | -0.010 | -0.011 | -0.013 | -0.017 | -0.011 | -0.021 | -0.013 | -0.008 | -0.015 | -0.014 | -0.015 | -0.011 | -0.024 | -0.020 | -0.022 | -0.018 | -0.012 | -0.025 | 0.063 | 0.415 | 0.189 | -0.012 | 0.031 | 0.215 | 0.863 | 0.879 | 0.995 | 0.994 | 0.998 | 0.973 | 1 |

**Supplementary Table S9** Primer used to investigate the regions of difference for differentiating *M. tuberculosis* into major lineages[40-43].

| | | | | Expected PCR product size (bp) | |
|---|---|---|---|---|---|
| Region of difference | Deletion size (bp) | Forward (F) and reverse (R) PCR primers | Annealing temperature (°C) | H37Rv | Deleted strain |
| RD105 | 3467 | F: GAGTCGTTGAGGGTGTTCATCA GCTCAGTC<br>R: CGCCAAGGCCGCATAGTCACGG TCG | 64 | 4252 | 785 |
| RD239 | 842 | F: GGCCAACATCGACCACCTACCC<br>R: ATCCTCGCTACCGGCACCTCAT | 64 | 1730 | 888 |
| RD750 | 790 | F: GTCGGCGGTCTGCTTCGTTCC<br>R: CCTGTCGGCCGGGTGTCTTTC | 64 | 1533 | 743 |
| Pks15/1 | 7 | F: GCAGGCGATGCGTCATGGGG<br>R: TCTTGCCCACCGACCCTGGC | 67 | 520 | 520* |
| TbD1 | 2155 | F: CTACCTCATCTTCCGGTCCA<br>R: CATAGATCCCGGACATGGTG | 65 | 484 | 484 |

*Differentiation was performed by Sanger sequencing.

**Supplementary Table S10** The accession numbers of 480 L1 isolates in this study are shown in the excel file**: Palittapongarnpim TS10 mTB 480 Indo Oceanic.xlsx**.

## References


1 Coll, F. *et al.* A robust SNP barcode for typing Mycobacterium tuberculosis complex strains. *Nat Commun* **5**, 4812, doi:10.1038/ncomms5812 (2014).

2 Comas, I. *et al.* Out-of-Africa migration and Neolithic coexpansion of Mycobacterium tuberculosis with modern humans. *Nat Genet* **45**, 1176-1182, doi:10.1038/ng.2744 (2013).

3 Manson, A. L. *et al.* Mycobacterium tuberculosis Whole Genome Sequences From Southern India Suggest Novel Resistance Mechanisms and the Need for Region-Specific Diagnostics. *Clin Infect Dis* **64**, 1494-1501, doi:10.1093/cid/cix169 (2017).

4 Hawkey, J. *et al.* ISMapper: identifying transposase insertion sites in bacterial genomes from short read sequence data. *BMC Genomics* **16**, 667, doi:10.1186/s12864-015-1860-2 (2015).

5 Zhang, J. *et al.* A first assessment of the genetic diversity of Mycobacterium tuberculosis complex in Cambodia. *BMC Infect Dis* **11**, 42, doi:10.1186/1471-2334-11-42 (2011).

6 Sasmono, R. T. *et al.* Heterogeneity of Mycobacterium tuberculosis strains in Makassar, Indonesia. *Int J Tuberc Lung Dis* **16**, 1441-1448, doi:10.5588/ijtld.12.0055 (2012).

7 Ismail, F. *et al.* Study of Mycobacterium tuberculosis complex genotypic diversity in Malaysia reveals a predominance of ancestral East-African-Indian lineage with a Malaysia-specific signature. *PLoS One* **9**, e114832, doi:10.1371/journal.pone.0114832 (2014).

8 Phyu, S. *et al.* Predominance of Mycobacterium tuberculosis EAI and Beijing lineages in Yangon, Myanmar. *J Clin Microbiol* **47**, 335-344, doi:10.1128/JCM.01812-08 (2009).

9 Douglas, J. T. *et al.* Characterization of the Manila family of Mycobacterium tuberculosis. *J Clin Microbiol* **41**, 2723-2726 (2003).

10 Sia, I. G. *et al.* Genotypic characteristics of Mycobacterium tuberculosis isolated from household contacts of tuberculosis patients in the Philippines. *BMC Infect Dis* **13**, 571, doi:10.1186/1471-2334-13-571 (2013).

11 Sun, Y. J. *et al.* Characterization of ancestral Mycobacterium tuberculosis by multiple genetic markers and proposal of genotyping strategy. *J Clin Microbiol* **42**, 5058-5064, doi:10.1128/JCM.42.11.5058-5064.2004 (2004).

12 Yorsangsukkamol, J. *et al.* Molecular analysis of Mycobacterium tuberculosis from tuberculous meningitis patients in Thailand. *Tuberculosis (Edinb)* **89**, 304-309, doi:10.1016/j.tube.2009.05.001 (2009).

13 Nguyen, V. A. *et al.* High prevalence of Beijing and EAI4-VNM genotypes among M. tuberculosis isolates in northern Vietnam: sampling effect, rural and urban disparities. *PLoS One* **7**, e45553, doi:10.1371/journal.pone.0045553 (2012).

14 Duong, D. A. *et al.* Beijing genotype of Mycobacterium tuberculosis is significantly associated with high-level fluoroquinolone resistance in Vietnam. *Antimicrob Agents Chemother* **53**, 4835-4839, doi:10.1128/AAC.00541-09 (2009).

15 Huang, S. F. *et al.* Association of Mycobacterium tuberculosis genotypes and clinical and epidemiological features - a multi-center study in Taiwan. *Infect Genet Evol* **12**, 28-37, doi:10.1016/j.meegid.2011.10.001 (2012).

16 Chang, J. R. *et al.* Clonal expansion of both modern and ancient genotypes of Mycobacterium tuberculosis in southern Taiwan. *PLoS One* **7**, e43018, doi:10.1371/journal.pone.0043018 (2012).

17 Chang, C. W., Wu, M. H., Chuang, P. C. & Jou, R. Characteristics of multidrug-resistant Mycobacterium tuberculosis in Taiwan: a population-based study. *Infect Genet Evol* **11**, 633-639, doi:10.1016/j.meegid.2011.01.021 (2011).

18 Rahim, Z. *et al.* Assessment of population structure and major circulating phylogeographical clades of Mycobacterium tuberculosis complex in Bangladesh suggests a high prevalence of

a specific subclade of ancient M. tuberculosis genotypes. *J Clin Microbiol* **45**, 3791-3794, doi:10.1128/JCM.01247-07 (2007).

19 Banu, S. *et al.* Molecular epidemiology of tuberculosis in rural Matlab, Bangladesh. *Int J Tuberc Lung Dis* **16**, 319-326, doi:10.5588/ijtld.11.0426 (2012).

20 Banu, S. *et al.* Multidrug-resistant tuberculosis in admitted patients at a tertiary referral hospital of Bangladesh. *PLoS One* **7**, e40545, doi:10.1371/journal.pone.0040545 (2012).

21 Storla, D. G. *et al.* Heterogeneity of Mycobacterium tuberculosis isolates in Sunamganj District, Bangladesh. *Scand J Infect Dis* **38**, 593-596, doi:10.1080/00365540600606465 (2006).

22 Gutierrez, M. C. *et al.* Predominance of ancestral lineages of Mycobacterium tuberculosis in India. *Emerg Infect Dis* **12**, 1367-1374, doi:10.3201/eid1209.050017 (2006).

23 Narayanan, S. *et al.* Genomic interrogation of ancestral Mycobacterium tuberculosis from south India. *Infect Genet Evol* **8**, 474-483, doi:10.1016/j.meegid.2007.09.007 (2008).

24 Shanmugam, S., Selvakumar, N. & Narayanan, S. Drug resistance among different genotypes of Mycobacterium tuberculosis isolated from patients from Tiruvallur, South India. *Infect Genet Evol* **11**, 980-986, doi:10.1016/j.meegid.2011.03.011 (2011).

25 Joseph, B. V. *et al.* Molecular epidemiology of Mycobacterium tuberculosis isolates from Kerala, India using IS6110-RFLP, spoligotyping and MIRU-VNTRs. *Infect Genet Evol* **16**, 157-164, doi:10.1016/j.meegid.2013.01.012 (2013).

26 Thomas, S. K. *et al.* Modern and ancestral genotypes of Mycobacterium tuberculosis from Andhra Pradesh, India. *PLoS One* **6**, e27584, doi:10.1371/journal.pone.0027584 (2011).

27 Singh, U. B. *et al.* Genetic biodiversity of Mycobacterium tuberculosis isolates from patients with pulmonary tuberculosis in India. *Infect Genet Evol* **7**, 441-448, doi:10.1016/j.meegid.2007.01.003 (2007).

28 Varma-Basil, M. *et al.* Comparison of spoligotyping, mycobacterial interspersed repetitive units typing and IS6110-RFLP in a study of genotypic diversity of Mycobacterium tuberculosis in Delhi, North India. *Mem Inst Oswaldo Cruz* **106**, 524-535 (2011).

29 Sharma, P. *et al.* Comparative study of genotypes of Mycobacterium tuberculosis from a Northern Indian setting with strains reported from other parts of India and neighboring countries. *Tuberculosis (Edinb)* **105**, 60-72, doi:10.1016/j.tube.2017.04.003 (2017).

30 Singh, J. *et al.* Genetic diversity and drug susceptibility profile of Mycobacterium tuberculosis isolated from different regions of India. *J Infect* **71**, 207-219, doi:10.1016/j.jinf.2015.04.028 (2015).

31 Desikan, P. *et al.* Genetic diversity of Mycobacterium tuberculosis isolates from central India. *Indian J Med Res* **143**, 481-486, doi:10.4103/0971-5916.184287 (2016).

32 Gupta, A., Kulkarni, S., Rastogi, N. & Anupurba, S. A study of Mycobacterium tuberculosis genotypic diversity & drug resistance mutations in Varanasi, north India. *Indian J Med Res* **139**, 892-902 (2014).

33 Devi, K. R. *et al.* Genetic Diversity of Mycobacterium tuberculosis Isolates from Assam, India: Dominance of Beijing Family and Discovery of Two New Clades Related to CAS1_Delhi and EAI Family Based on Spoligotyping and MIRU-VNTR Typing. *PLoS One* **10**, e0145860, doi:10.1371/journal.pone.0145860 (2015).

34 Malla, B. *et al.* First insights into the phylogenetic diversity of Mycobacterium tuberculosis in Nepal. *PLoS One* **7**, e52297, doi:10.1371/journal.pone.0052297 (2012).

35 Tanveer, M. *et al.* Genotyping and drug resistance patterns of M. tuberculosis strains in Pakistan. *BMC Infect Dis* **8**, 171, doi:10.1186/1471-2334-8-171 (2008).

36 Viegas, S. O. *et al.* Molecular diversity of Mycobacterium tuberculosis isolates from patients with pulmonary tuberculosis in Mozambique. *BMC Microbiol* **10**, 195, doi:10.1186/1471-2180-10-195 (2010).

37 Stavrum, R. *et al.* Increased level of acute phase reactants in patients infected with modern Mycobacterium tuberculosis genotypes in Mwanza, Tanzania. *BMC Infect Dis* **14**, 309, doi:10.1186/1471-2334-14-309 (2014).

38 Mbugi, E. V. *et al.* Genetic diversity of Mycobacterium tuberculosis isolated from tuberculosis patients in the Serengeti ecosystem in Tanzania. *Tuberculosis (Edinb)* **95**, 170-178, doi:10.1016/j.tube.2014.11.006 (2015).

39 Mbugi, E. V. *et al.* Mapping of Mycobacterium tuberculosis Complex Genetic Diversity Profiles in Tanzania and Other African Countries. *PLoS One* **11**, e0154571, doi:10.1371/journal.pone.0154571 (2016).

40 Tsolaki, A. G. *et al.* Genomic deletions classify the Beijing/W strains as a distinct genetic lineage of Mycobacterium tuberculosis. *J Clin Microbiol* **43**, 3185-3191, doi:10.1128/JCM.43.7.3185-3191.2005 (2005).

41 Constant, P. *et al.* Role of the pks15/1 gene in the biosynthesis of phenolglycolipids in the Mycobacterium tuberculosis complex. Evidence that all strains synthesize glycosylated p-hydroxybenzoic methyl esters and that strains devoid of phenolglycolipids harbor a frameshift mutation in the pks15/1 gene. *J Biol Chem* **277**, 38148-38158, doi:10.1074/jbc.M206538200 (2002).

42 Brosch, R. *et al.* A new evolutionary scenario for the Mycobacterium tuberculosis complex. *Proc Natl Acad Sci U S A* **99**, 3684-3689, doi:10.1073/pnas.052548299 (2002).

43 Gagneux, S. *et al.* Variable host-pathogen compatibility in Mycobacterium tuberculosis. *Proc Natl Acad Sci U S A* **103**, 2869-2873, doi:10.1073/pnas.0511240103 (2006).
